# The Multiple Functions of the Nucleolus in Plant Development, Disease and Stress Responses

**DOI:** 10.3389/fpls.2018.00132

**Published:** 2018-02-09

**Authors:** Natalia O. Kalinina, Svetlana Makarova, Antonida Makhotenko, Andrew J. Love, Michael Taliansky

**Affiliations:** ^1^Branch of the Shemyakin-Ovchinnikov Institute of Bioorganic Chemistry of the Russian Academy of Sciences, Moscow, Russia; ^2^The James Hutton Institute, Dundee, United Kingdom

**Keywords:** the nucleolus, plant development, plant stress responses, virus, plant–pathogen interactions

## Abstract

The nucleolus is the most conspicuous domain in the eukaryotic cell nucleus, whose main function is ribosomal RNA (rRNA) synthesis and ribosome biogenesis. However, there is growing evidence that the nucleolus is also implicated in many other aspects of cell biology, such as regulation of cell cycle, growth and development, senescence, telomerase activity, gene silencing, responses to biotic and abiotic stresses. In the first part of the review, we briefly assess the traditional roles of the plant nucleolus in rRNA synthesis and ribosome biogenesis as well as possible functions in other RNA regulatory pathways such as splicing, nonsense-mediated mRNA decay and RNA silencing. In the second part of the review we summarize recent progress and discuss already known and new hypothetical roles of the nucleolus in plant growth and development. In addition, this part will highlight studies showing new nucleolar functions involved in responses to pathogen attack and abiotic stress. Cross-talk between the nucleolus and Cajal bodies is also discussed in the context of their association with poly(ADP ribose)polymerase (PARP), which is known to play a crucial role in various physiological processes including growth, development and responses to biotic and abiotic stresses.

## Plant nucleolar organization

The nucleolus is the largest and most prominent domain in the eukaryotic interphase cell nucleus. Nucleoli vary in size in different cells, for example in small cells like yeast they are <1 μm diameter, whereas in larger cells such as pea they are >10 μm in diameter (Shaw, 2015). The nucleolus is a dynamic membrane-less structure whose primary function is ribosomal RNA (rRNA) synthesis and ribosome biogenesis. However, there is mounting evidence that the nucleolus is also implicated in many other aspects of cell biology, such as differentiation, cell cycle regulation, growth and development, senescence, gene silencing, telomerase activity, responses to biotic and abiotic stresses, and biogenesis of various ribonucleoprotein (RNP) particles (Olson and Dundr, 2005; Boisvert et al., 2007; Hiscox, 2007; Sirri et al., 2008; Greco, 2009; Taliansky et al., 2010; Stepinski, 2014; Brighenti et al., 2015; Lafontaine, 2015; Weis et al., 2015b).

**Figure 1:**
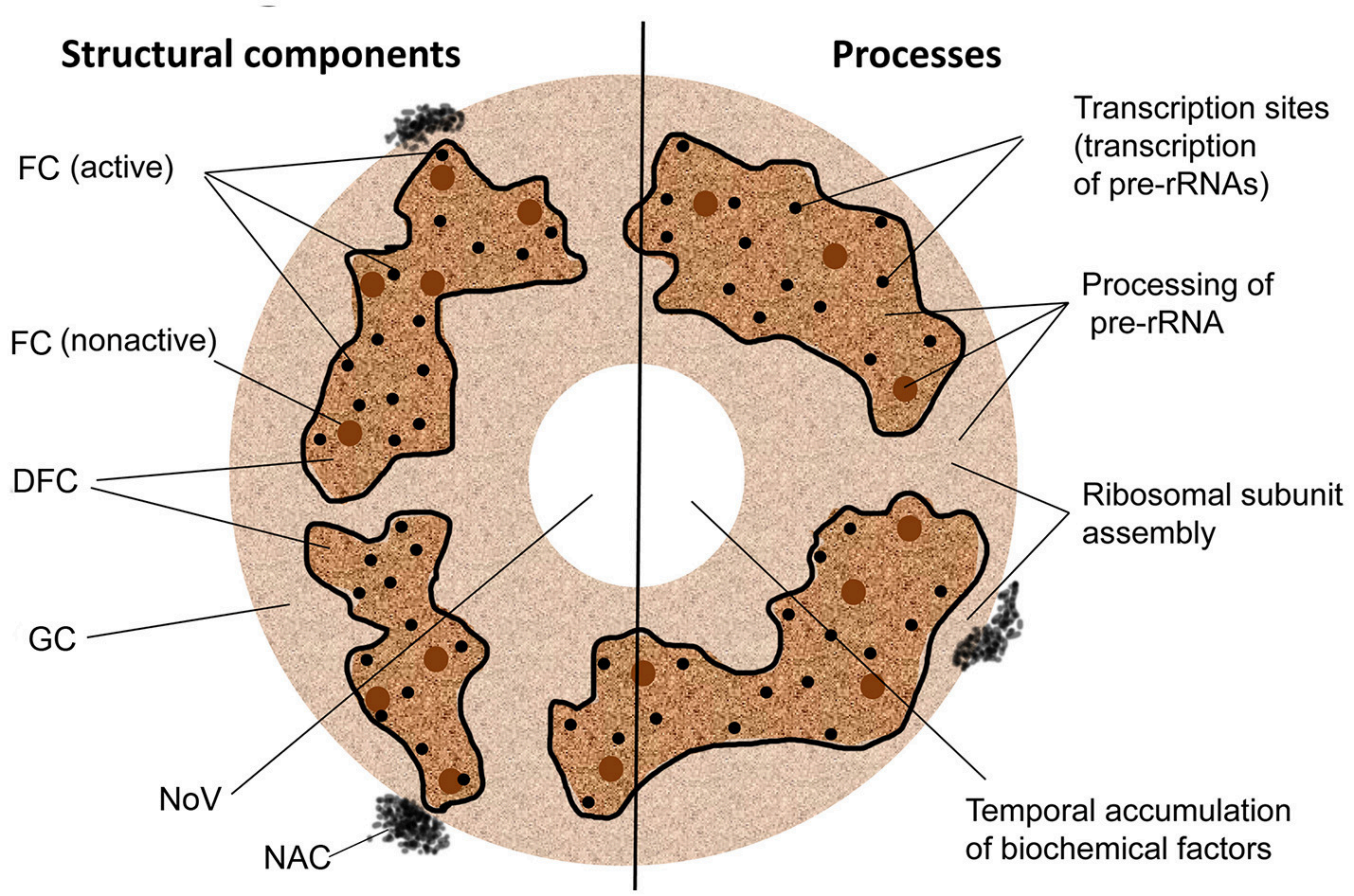
Structural and functional domains of the nucleolus. FC, fibrillar center; DFC, the dense fibrillar component; GC, the granular component; NoV, nucleolar vacuole; NAC, nucleolus-associated chromatin. Nucleolonema is encircled with black lines.

The plant nucleolus has a well-defined architecture with prominent functional compartments such as fibrillar centers (FC), the dense fibrillar component (DFC), the granular component (GC), nucleolar chromatin, nucleolar vacuoles, and nucleolonema (Figure 1; Stepinski, 2014). It is largely formed of proteins (85–90%) and RNA (5–10%), with rDNA comprising a minor component (Gerbi and Borovjagin, 1997; Shaw and Brown, 2012).

Interestingly the DFC and FC nucleolar components are typically organized into an important nucleolar substructure called the nucleolonema, which is composed of a DFC matrix punctuated with spherical or tubular FCs, and may also contain FC condensed chromatin and harbor rDNA. This structure has several functional domains such as rDNA transcription, transcript processing and ribosome assembly zones, which are consistent with the activities associated with DFC and FC (Yano and Sato, 2002; Sato et al., 2005).

Plant FCs are assumed to be the assembly sites of complexes containing transcription-associated factors which can either be ready for transcription or be in an inactive state (de Carcer and Medina, 1999; González-Camacho and Medina, 2006). Plant FCs also contain rDNA, which are not yet engaged in transcription but may later be deployed to this process in specific circumstances (Shaw, 1996; McKeown and Shaw, 2009). The number and sizes of FCs depend on the cell cycle phases: cells in G1 phase normally possess much fewer numbers of FCs than those at the G2 phase (Grummt, 2003; González-Camacho and Medina, 2006).

In plant nucleoli the DFC occupies the majority of the nucleolar volume (up to 70%) and provides an environment for transcription of precursor rRNAs (pre-rRNAs); a process which can occur simultaneously at multiple sites (200–400) within this region. The produced pre-rRNAs subsequently undergo further processing in the DFC and then in the associated GC. For example, localization studies of pre-rRNAs and different small nucleolar RNAs (snoRNAs) and proteins have elucidated that early and late pre-rRNA cleavage events can occur in the DFC and GC, respectively, suggesting a vectorial model for the production and maturation of rRNAs (Brown and Shaw, 1998). It is thought that in the GC the final steps of assembly of small and large ribosomal subunits from mature rRNAs and ribosomal proteins occurs, and that the GC could participate in the transit of the assembled ribosomes through the extranucleolar nucleoplasm to the cytoplasm (Shaw et al., 1995; Shaw, 1996).

Plant nucleoli also usually contain so called nucleolar cavities or vacuoles (NoV) often located in the central part of the nucleolus (Figure 1; Brown and Shaw, 1998; Shaw and Brown, 2004). Although the function of the NoVs is currently unknown, it could be suggested that these structures may be regions of temporal sequestration and storage of some biochemical factors such as elements of the ubiquitin-proteasome system (Stepinski, 2012), snoRNAs (snoRNPs) and spliceosomal small nuclear RNAs (snRNAs/snRNPs) (Beven et al., 1996; Lorković and Barta, 2008), which may be released into the nucleoplasm depending on specific physiological requirements incurred during stress responses or at particular developmental stages (Mineur et al., 1998).

Chromatin clusters which are often associated with the nucleolus (nucleolus-associated chromatin domains, NADs) primarily have a heterochromatic nature, and comprise sub-telomeric regions, transposable elements (TEs), and largely inactive protein-coding genes (Pontvianne et al., 2016). However, NADs also include active rRNA genes, which are typically arranged in tandem DNA arrays, (known as nucleolus organizer regions, or NORs). In Arabidopsis, NORs are located on the left arms of chromosomes 2 and 4 (NOR2 and NOR4, respectively; Chandrasekhara et al., 2016). In the wild type plants only NOR4 and the adjacent entire short arm of chromosome 4 were shown to be associated with the nucleolus (Pontvianne et al., 2016). In contrast NOR2 and its neighboring region in chromosome 2, were excluded from the nucleolus and had inactive rRNA genes (Chandrasekhara et al., 2016; Pontvianne et al., 2016); this may suggest that although NOR2 may have structural similarity to NOR4, its activity may differ depending on the experimental/environmental conditions. Interestingly there are indications that NOR2 and NOR4 may share some regulatory mechanisms as suggested by null mutants for the *NUCLEOLIN 1* gene (encoding one of the major nucleolar proteins, nucleolin 1–see below). In these null mutants both NOR4 and NOR2 localized to the nucleolus, and the NOR2 rRNA genes which are usually silenced during development in wild-type leaves, became active (Pontvianne et al., 2010). Among the genes found to be localized to the nucleolus were functional genes, tRNA genes, and pseudogenes (Pontvianne et al., 2016). Since RNA polymerase II (Pol II) is not present in the nucleolus, it can be assumed that those NAD-genes which are normally transcribed by Pol II would likely not be expressed; this may constitute a novel mechanism of gene expression regulation.

NORs constitute sites on metaphase chromosomes where nucleoli become organized during reinitiation of transcription in postmitotic cells as they enter interphase. After cell division, nucleoli are reconstituted on NOR sites that contain rDNA genes which were transcriptionally active during the previous interphase but remained comparatively decondensed during mitosis (Heliot et al., 1997; Mais et al., 2005; Prieto and McStay, 2008). The newly organized nucleoli are rebuilt from rDNA gene products, such as primary pre-ribosomal RNAs undergoing different steps of processing, constituents of transcriptional and processing machineries which include U3 snoRNA, and major nucleolar proteins such as nucleolin, fibrillarin, Nop52 and B23. These components, which are derived from the previous interphase nucleoli, first form perichromosomal compartments, then prenucleolar bodies and, finally culminate in the formation of nucleolus-derived foci (Dundr and Olson, 1998; Hernandez-Verdun, 2011; Carron et al., 2012). At the end of mitosis (late telophase) the formation of one or more nucleoli at each active NOR occurs, and these small nucleoli often fuse together to form a single nucleolus (this frequently occurs in plant cells) as interphase progresses (Shaw and Jordan, 1995).

## The nucleolus and ribosome production

The major activities of the nucleolus are associated with ribosome production (Figure 1). In the nucleolus, RNA polymerase I (RNA Pol I) mediates the transcription of the pre-rRNA, which takes the form of 45S rRNA. This pre-rRNA can either be co- or post-transcriptionally processed by snoRNPs (small nucleolar ribonucleoproteins) to produce 5.8S, 18S, and 28S rRNAs (Nazar, 2004; Russell and Zomerdijk, 2005) which may also be 2′-O-methylated and pseudouridinylated (Matera et al., 2007). After processing, suitable rRNA species assemble with ribosomal proteins into small and large pre-ribosomal subunits (Fromont-Racine et al., 2003) which are exported separately to the cytoplasm where they are modified further to form mature 60S and 40S ribosome subunits. These three activities of the nucleolus (pre-rRNA synthesis, processing, and ribosomal RNP assembly) are well consistent with its FC, DFC, and GC derived “tripartite” internal structure mentioned above. Indeed, pre-rRNA appears to be transcribed from rDNA in the FC or at its border with the DFC. For example, FCs are enriched in RNA Pol I machinery components (such as UBF), and the DFC contains factors involved in pre-rRNA processing, such as fibrillarin, snoRNAs, snoRNP proteins and Nop58. The FC and DFC are both surrounded by the GC, where pre-ribosome subunits are assembled (Boisvert et al., 2007; Sirri et al., 2008; Boulon et al., 2010).

## Protein composition and plurifunctionality of the nucleolus

The three most abundant and major rRNA-associated nucleolar proteins involved in ribosome biogenesis are fibrillarin, nucleolin, and B23. Fibrillarin is a key component of box C/D snoRNP particles and has methyltransferase activity which directs 2′-O-ribose methylation of rRNA and spliceosomal snRNAs, and is required for pre-rRNA processing and splicing of snoRNA (Warner, 1990; Tollervey et al., 1993). Nucleolin plays an important role in regulating chromatin structure-mediated rDNA transcription and processing of pre-rRNA (Ginisty et al., 1998; Roger et al., 2003; Pontvianne et al., 2007), the assembly of ribosome particles and their nucleocytoplasmic transport (Bouvet et al., 1998). B23 (nucleophosmin) plays a crucial role in maintaining nucleolar structure, rDNA transcription, rRNA maturation, ribosome assembly and export (Murano et al., 2008). While much is known about the ribosome biogenesis functions of these proteins, it is becoming clear that they are also involved in a broad range of processes other than ribosome synthesis. Moreover, accumulating evidence shows that many other proteins and RNAs completely unrelated to ribosome production are present in the nucleolus. Protein and RNA localization studies and comprehensive proteomic analyses of both human and plant nucleoli enabled identification of these macromolecules (Andersen et al., 2002; Pendle et al., 2005; Ahmad et al., 2009; Lewandowska et al., 2013). Thus, many non-conventional functions have been attributed to the nucleolus (Pederson, 1998; Olson and Dundr, 2005). In plants, these functions include surveillance (nonsense-mediated decay) of mRNA, metabolism, modifications, assembly, or transport of various small nuclear and nucleolar RNAs (snRNAs and snoRNAs) and regulatory RNAs (siRNAs and miRNAs) (Pendle et al., 2005; Brown and Shaw, 2008; Kim et al., 2009, 2010; Shaw and Brown, 2012; Pontes et al., 2013), interactions with DNA and RNA viruses (see for reviews Hiscox, 2007; Greco, 2009; Taliansky et al., 2010) and other pathogens (Jones et al., 2009; Leonelli et al., 2011; Stam et al., 2013; Petre et al., 2015; Boevink et al., 2016), stress sensing, signaling and defense pathways (Lewandowska et al., 2013), DNA damage responses (Yoshiyama et al., 2014; Yoshiyama, 2016); Manova and Gruszka (2015) and developmental control (Weis et al., 2015a). These non-canonical functions of the nucleolus will be discussed in detail below (see Tables 1, 2).

**Table 1:** Role of selected proteins in plant development and stress responses.

**Protein**	**Function / process**	**Loss-of-function / gain-of-function phenotype**	**References**
atNucleolin	Various steps of ribosome biogenesis	Gene disruption (Δ*AtNuc-L1-1* plants): reduced growth rate, prolonged life, bushy growth, pointed leaves, and defective vascular patterns and pod development Induction of AtNuc-L1-1: growth resumption	Kojima et al., 2007
atBRX-1-1 and atBRX-1-2	Maturation of the large pre-60S ribosomal subunit	*brx1-1brx1-2:* delay in development *1brx1-2*: pointed leaves	Weis et al., 2015a
atRPS18A; atRPS13A; atRPS5A; atRPL24B	Ribosomal proteins	Gene disruptions: phenotypes are similar to those observed for Δ*AtNuc-L1-1* plants	Van Lijsebettens et al., 1994; Ito et al., 2000; Weijers et al., 2001; Nishimura et al., 2005
atRPL23a	Ribosomal protein;ribosome biogenesis	*RPL23aA* RNAi: growth delay, irregularities in morphology of leaves, roots, phyllotaxy and vasculature, and loss of apical dominance	Degenhardt and Bonham-Smith, 2008
RBFs, ribosome biogenesis factors	Pre-rDNA transcription, pre-rRNA processing, modification, folding, and assembly with RPs	Gene disruptions: infertility, embryo lethality, impaired growth and gametophyte development, aberrant cotyledon, leaf and root development	Weis et al., 2015b
atTHAL: SAS10/C1D family protein	Processing of precursor rRNAs, and expression of the major rDNA variant (*VAR1)*	*thal-1* and *thal-2*: lethal early in reproductive development; enlarged nucleoli in arrested embryos THAL overexpression: multiple nucleoli	Chen et al., 2016
osNMD3	Non-sense mediated decay; 60S pre-ribosome export and maturation	Overexpression of *osNMD3^Δ*NLS*^*: dwarfism and decrease in the internode length	Shi et al., 2014
atSGP1/2 and bnMAP4Ka	Homologous to fission yeast spg1p and sid1p, respectively - septation initiation network (SIN)	Overexpression in yeast complements mutant spg1-B8 and sid1-239 proteins and induces multisepta in wild-type yeast, suggesting the existence of plant SIN-related cell cycle network	Champion et al., 2004
MAGO and Y14	Components of EJC: nonsense mediated decoy	RNAi: male infertility, defects in pollen grain maturation, spermatogenesis, floral and vegetative growth and stamen development; defects in root, shoot and seed development	Chen et al., 2007; He et al., 2007; Park et al., 2009; Boothby and Wolniak, 2011; Gong and He, 2014.
RID1	a DEAH-box RNA helicase; splicing	*rid1-1*: abnormalities in meristem maintenance and leaf and root morphogenesis	Ohtani et al., 2013
TERT	Catalytic subunit of telomerase; interacts with dyskerin	TERT activity is developmentally regulated in plants (high in reproductive organs but low in vegetative tissues)	Procházková Schrumpfová et al., 2016
STRS1 and STRS2	DEAD-box RNA helicases; negative regulators of stress-induced gene expression	*strs* mutants: enhanced tolerance to salt, osmotic and heat stress *STRS* overexpression: diminished tolerance to salt and heat stress	Khan et al., 2014
atRab 28 LEA	unknown	Overexpression: increased leaf and root areas, higher relative water content and reduced chlorophyll loss when grown under osmotic stress	Amara et al., 2013
atREN1	Strongly homologous to the heat shock transcription factor gene *HSFA5*	*ren*: abnormalities in male gametophyte and pollen grain development, and perturbed heat stress responses	Renák et al., 2014
Coilin	The signature protein of CB; essential for CB formation and function	RNAi: enhanced salt stress tolerance.	Love et al., 2017
Poly (ADP-ribose) polymerase (PARP)	PARP modifies the function of a variety of nuclear “target” proteins by attaching chains of ADP ribose them and itself	*atPARP2* overexpression: diminished incidence of DNA nicks at high H_2_O_2_ concentration and increased incidence of DNA nicks at low H_2_O_2_ concentration atPARP1/PARP2 knock down: enhanced tolerance to drought, oxidative and high light stress	Reviewed in Briggs and Bent, 2011
SOG1	Functional analog of animal p53: master regulator of DNA damage response (DDR) including stimulation of transcriptional response, cell cycle arrest and PCD	*sog1-1*: increased resistance of root growth to zeocin; no cell cycle arrest and PCD in response to DNA double-strand breaks (DSB)	Yoshiyama et al., 2014; Yoshiyama, 2016
RMI2 and RTEL1	Stabilization of plant 45S rDNA repeats	*rmi2-2 rtel1-1*: male infertility	Röhrig et al., 2016
TDP1	Tyrosyl DNA phosphodiesterase - DNA repair	*tdp1*: dwarfism, diminished cell number, developmental cell death (Arabidopsis) RNAi: reduced cell division, perturbed plant growth and early leaf senescence; impaired rRNA processing and ribosome biogenesis and disruption of the nucleolus (*M. truncatula*)	Lee et al., 2010; Donà et al., 2013

**Table 2:** Selected plant pathogen-nucleolar interactions.

**Pathogen**	**Non-host factor**	**Host factor**	**Function**	**References**
Groundnut rosette virus (GRV, umbravirus)	ORF3	fibrillarin	Association required for long-distance virus movement	Canetta et al., 2007; Kim et al., 2007a,b
Potato leaf roll virus (PLRV, polerovirus)	Capsid protein (CP) and CP read-through protein	fibrillarin	Association required for long-distance virus movement	Haupt et al., 2005; Kim et al., 2007b
Bamboo mosaic virus (BaMV, potexvirus)- associated satRNA (satBaMV)	p20 satBaMV	fibrillarin	Association required for long-distance virus movement	Chang et al., 2016
Rice stripe virus (RSV, tenuvirus)	p2 protein (silencing suppressor protein)	fibrillarin	Association required for long-distance virus movement	Zheng et al., 2015
Potato virus A (PVA, potyvirus)	VPg domain of nuclear inclusion protein a (NIa)	fibrillarin	Depletion of fibrillarin reduces accumulation of PVA; this may operate through association of VPg with fibrillarin	Rajamäki and Valkonen, 2009
Poa semilatent virus(PSLV, hordeivirus)	TGBp1 (Triple gene block protein 1)	fibrillarin	Functions of this association remain to be elucidated	Semashko et al., 2012
*Barley stripe mosaic* virus (BSMV, hordeivirus)	TGBp1 (Triple gene block protein 1)	fibrillarin	Association required for cell-to-cell virus movement	Li et al., 2017
Potato virus A (PVA) and *turnip mosaic* virus (TuMV) (potyviruses)	VPg	S6K (protein S6 kinase)	Silencing of the S6K gene in *N. benthamiana* decreases accumulation of PVA and TuMV	Rajamäki et al., 2017
Cucumber mosaic virus (CMV, cucumovirus)	2b, silencing suppressor	Argonaute4	Functions of this association remain to be elucidated	González et al., 2010; Du et al., 2014
Alfalfa mosaic virus (AlMV, alfamovirus)	CP	ILR3 (transcription factor of a basic helix–loop–helix family of TFs)	The AlMV CP–ILR3 interaction leads to activation of plant hormone responses, which forms a hormonal balance optimal for plant viability and virus production	Aparicio and Pallás, 2017
Tomato bushy stunt virus (TBSV, tombusvirus)	P19 (silencing suppressor protein)	ALY proteins	ALY proteins may interfere with the silencing suppressor activity of P19, which could constitute a novel antivirus defense response	Canto et al., 2006
*Globodera pallida* (potato cyst nematode)	two protein effectors 22E10 and 13G11		Suppresses host defense	Jones et al., 2009
*Phytophtora infestans* (oomycete plant pathogen)	effector Avr3a protein	E3 ligase CMPG1	Association regulates host resistance	Bos et al., 2010; Gilroy et al., 2011
*Hyaloperonospora arabidopsidis* (obligate biotrophic oomycete pathogen)	HaRxL44	Mediator subunit 19a (MED19a)	Pathogen effector modulates host transcription to enhance invasion	Caillaud et al., 2013
*Hyaloperonospora arabidopsidis*	ATR13 Emco5	RPP13-Nd	interaction triggers hypersensitive response which limits pathogen spread	Leonelli et al., 2011
*P. infestans*	Pi04314	phosphatase 1 catalytic (PP1c) isoforms	Promotes late blight disease by attenuating transcription of host plant defense genes	Boevink et al., 2016

## New nucleolar functions in RNA regulatory pathways

### Exon junction complex (EJC)

The most striking finding from the proteomic analysis of the Arabidopsis nucleolus is that this sub-nuclear organelle comprises six proteins of the EJC: UAP56, MAGO, ALY/REF, RNPS1, Y14, and the translation initiation factor eIF4A-III, whereas in animals these proteins localize to cytoplasmic processing bodies (Pendle et al., 2005). Components of EJC mark splice junctions in mRNAs after mRNA splicing and play key roles in various post-splicing processes such as mRNA export from the nucleus to its cytoplasmic location, and the nonsense-mediated mRNA decay (NMD) pathway of mRNA surveillance (Dreyfuss et al., 2002; Maquat, 2004). The NMD surveillance system recognizes and degrades aberrant (truncated) mRNAs that contain a premature termination codon. It has been shown that in plants there is a greater abundance of aberrant mRNAs in the nucleolus, while in the nucleoplasm fully spliced products are more abundant. Moreover, direct correlation between aberrant mRNA accumulation in the nucleolus and their NMD-mediated turnover has been demonstrated using Arabidopsis *upf* mutants, which are known to be impaired in NMD, whereby mRNAs that are typically degraded by NMD will accumulate in nucleoli (Kim et al., 2009). This suggests that the plant nucleolus is directly involved in recognizing aberrant mRNAs and NMD. A possible role of the EJC components in plant development is discussed later in the chapter.

### Novel small nucleolar RNAs

With regard to snoRNAs, they form an abundant class of non-coding small RNAs that guide 2 main types of modifications of other RNAs, such as rRNAs, tRNAs, and snRNAs (Love et al., 2017). The C/D box snoRNAs are associated with fibrillarin (methyltransferase) and other additional proteins to form snoRNPs which direct 2′-O- methylation of RNA targets. Whereas, the H/ACA box snoRNAs forms a complex with dyskerin (pseudouridine synthase) which guides pseudouridylation of specific nucleotides. Another group of small RNAs which are structurally related to snoRNAs are small Cajal bodies-specific RNAs (scaRNAs), and these are found in abundance in Cajal bodies (CBs), sub-nuclear structures functionally and physically connected to the nucleolus (Love et al., 2017). scaRNAs contain either or both of the boxes together and modify certain snRNAs. Using an RNomics approach on Arabidopsis, 188 different scaRNA/snoRNA genes and 294 scaRNA/snoRNA gene variants were identified. In addition to snoRNA and scaRNAs, some novel “orphan” snoRNAs have also been found which do not have complementarity to rRNA or snRNAs but are expressed (e.g., Kim et al., 2010). Orphan snoRNAs have previously been found in other eukaryotes, and bioinformatic analysis of possible mRNAs which could be targets of orphan human snoRNAs revealed a potential connection with genes that are alternatively spliced; suggesting a function in regulating alternative splicing (Bazeley et al., 2008). Thus, it is possible that in plants orphan snoRNAs (besides modifying rRNAs and snRNAs) may target mRNA, which could affect gene regulation and influence plant development and growth. Another potential activity of snoRNAs may be attributed to the mechanism by which snoRNA can be processed to microRNAs (miRNAs) in human cells: DICER can process box H/ACA snoRNA to produce small RNAs which in association with Argonaute proteins cause depletion of target gene expression (Ender et al., 2008). It is thus intriguing to speculate whether these novel snoRNA functions described for other organisms also occur in plants and to what extent they could control plant growth and development; potential pathways which warrant future research.

### Gene silencing pathways and small RNAs

In addition to rRNAs, tRNAs, snRNAs, and snoRNAs, several other classes of small non-coding RNAs (small ncRNAs or sRNAs), namely silencing RNAs, have been implicated in regulatory functions in eukaryotes. Silencing RNAs constitute an exquisite and complex mechanism which are required for controlling plant development, determining epigenetic modifications (e.g., histone and DNA methylation) and defense against viruses (Mallory and Vaucheret, 2010), for example. The major types of sRNAs include microRNA (miRNA), natural cis-antisense siRNA (natsiRNA), trans-acting small interfering RNA (ta-siRNA), and heterochromatic small interfering RNA (hc-siRNA) (Mallory and Vaucheret, 2010). These RNA species effectively regulate various transcriptional and post-transcriptional gene silencing (TGS and PTGS) pathways by modulating mRNA production or degradation. These pathways are invoked by the presence of aberrant mRNA structures (such as RNA with hairpin loops or double stranded RNA (dsRNA), which may arise for instance from endogenous genes). Each pathway typically starts with the conversion of aberrant RNAs into dsRNA (if not already double stranded) by viral or endogenous RNA-dependent RNA polymerases (RDRs) (Mallory and Vaucheret, 2010) before their cleavage into 21–24 nucleotide (nt) dsRNA duplexes by specific DICER or DICER-like (for plants) enzymes (DCL). The sRNA duplexes are unraveled, with one strand binding an ARGONAUTE (AGO) protein, which then targets the RNA for cleavage, and mediates repression of translation or the establishes epigenetic modifications (Vaucheret, 2008).

Arabidopsis contains four DCLs, 10 AGOs, and six RDRs that operate in concert with various sRNAs in different combinations, forming a complex variety of silencing pathways. How unique pathways are determined for each individual sRNA is generally unknown. Recent localization studies have indicated that many proteins involved in the miRNA, hc-siRNA and ta-siRNA silencing pathways accumulate within sub-nuclear structures in the nucleolar periphery (Pontes et al., 2013). Cytological analysis of these structures indicated that these may be CBs or CB-related structures, which suggests that CBs may be a site for assembly, re-cycling and storage of RNA silencing components, and also a site for specific sRNA silencing pathways (Pontes et al., 2006, 2013). However, in mutant Arabidopsis plants which contain no conventional CBs, changes in siRNA accumulation or in DNA methylation patterns have not been detected. It can therefore be speculated that RNA silencing functions may still be fulfilled by other multiple CB-related bodies present in eukaryotic cells, e.g., pre-CB structures which may be produced by some CB components at the early stages of their formation (Love et al., 2017).

CBs (as well as CB-related bodies) are dynamic structures, with major roles in RNA metabolism and formation of RNPs involved in transcription, splicing and ribosome biogenesis, and which are closely associated with the nucleolus. This, therefore, suggests a role for the nucleolus in RNA silencing pathways (Pontes and Pikaard, 2008). Indeed, some mature and precursor miRNAs are enriched in mammalian cell nucleoli (Politz et al., 2009; Scott et al., 2009). There is also cross-talk between snoRNAs and miRNA precursors, in which snoRNA precursors may be processed into some miRNAs, which may retain snoRNA features (Saraiya and Wang, 2008; Ono et al., 2011). In addition, sRNAs derived from snoRNAs were reported to associate with the AGO proteins of RNA silencing pathways in both Arabidopsis and animals (Taft et al., 2009), and it was observed that sRNAs derived from a human snoRNA could reduce expression of target genes (Ender et al., 2008). A challenge for future research is to give further insights into the precise molecular functions localized within the nucleolus and CBs (CB-related bodies) in regulating miRNA and siRNA pathways.

## Plant growth and development

**Figure 2:**
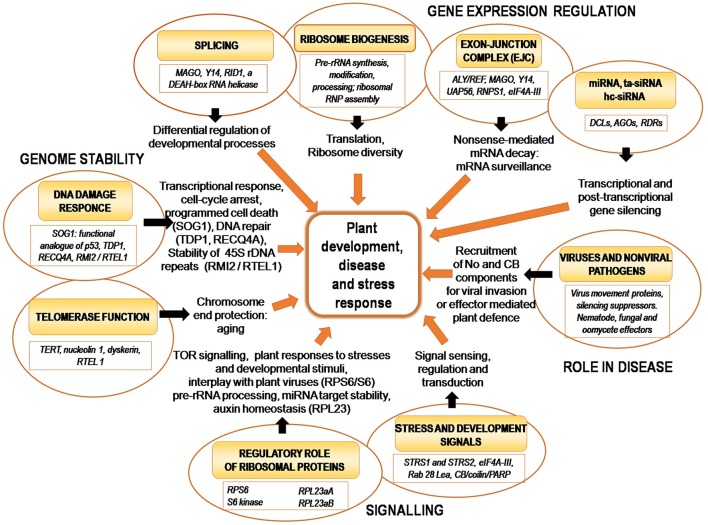
The role of the nucleolus and its molecular activities in regulating plant physiology.

Nucleolus-related ribosome production, spliceosome formation, gene expression regulation (e.g., transcriptional/post-transcriptional gene silencing), mRNA surveillance (EJC-mediated intron-based NMD pathway) and telomere maintenance (with links to aging) can be expected to play essential roles in plant growth and development. Indeed, several recent reports provide emerging evidence that these nucleolar activities are involved in various developmental processes. Since these data will be discussed in more detail in other papers of this Research Topic, this section on developmental regulation will only briefly cover these aspects, with a later emphasis on functional implications of nucleolar responses to pathogens and stress described further below (Tables 1, 2, Figure 2).

### Ribosome biogenesis and growth and development

The varied impacts of ribosome biogenesis on plant growth and development are illustrated in Table 1.

Two Arabidopsis proteins atBRX-1-1 and atBRX-1-2, which are highly similar in sequence, are mainly localized to the nucleolus and are implicated in maturation of the large pre-60S ribosomal subunit (Weis et al., 2015a). Plant lines deficient in both these factors showed significant developmental delays, and also pointed leaves were observed in the *brx1-2* mutant. Taken together this suggests a strong link between plant development and ribosome biogenesis.

Nucleolin is an abundant multifunctional nucleolar protein involved in various stages of ribosome biogenesis. Disruption of its gene in Arabidopsis (AtNuc-L1) led to reduced pre-rRNA processing and resulted in prolonged life, reduced growth rate, pointed leaves, bushy growth, and defective development of vascular patterns and pods, which are similar to those phenotypes reported for several RP gene mutants (Kojima et al., 2007). In contrast, induction of *AtNuc-L1* expression with glucose normalized plant growth. The reduced growth rate of nucleolin-deficient plants was presumably caused by reduced cell division due to a shortage of ribosomes. These data suggest that the rates of ribosome production in meristem tissues may have a significant effect on growth and plant architecture.

Like RPs, many other ribosome biogenesis factors (RBFs) are also involved in plant developmental processes (Weis et al., 2015b; Table 1, Figure 2). It is tempting to speculate that RBFs may take part in some specific modifications of RPs and rRNAs, which may facilitate remodeling of ribosome pools in response to developmental stimuli and environmental conditions (Lafontaine, 2015; Weis et al., 2015b). Such ribosome reprogramming may be closely related to nucleolar organization. In this respect, it is worth noting that, for example, the Arabidopsis THAL protein belonging to the SAS10/C1D family is involved in the processing of precursor rRNAs, specifically regulating expression of the major rDNA variant (*VAR1*). It was found that defects in *THAL* significantly increase nucleolar size in arrested embryos (Chen et al., 2016). On the other hand, *THAL* overexpression results not only in recovery of *VAR1* expression but also promotes formation of multiple nucleoli per nucleus, possibly linking changes in nucleolar organization with regulation of ribosome biogenesis.

Changes in ribosome biogenesis may also affect global protein synthesis which would inevitably affect plant growth and development. NMD3 is a nucleo-cytoplasmic shuttling protein which has been previously characterized as a component of the NMD pathway. It is also involved in transport and maturation of large ribosomal (60S) subunits (Shi et al., 2014). In rice, overexpression of an NMD3 mutant which contains a deleted nuclear localization site, was found to be retained in the cytoplasm and produced abnormalities in plant growth and development (dwarfism and decrease in the internode length, grain size and weight); these effects are possibly due to changes in ribosome biogenesis and consequent decreases in mRNA translation efficiency (Shi et al., 2014).

It is also worth noting that expression of many RP genes are controlled and activated by the target of rapamycin (TOR), a master cell cycle regulator. Plants overexpressing TAP46, an important factor of the TOR signaling network, demonstrated significant increases in production of some RPs (Weis et al., 2015b), corroborating the functional cross-talk between ribosome biogenesis and plant development and cell cycle.

### Plant nucleolus and cell cycle

Cytokinesis is the final phase of the cell cycle when the cell is divided into two daughter cells via formation of a cell plate between them, which is later converted into a proper cell wall. Generation of the cell plate (septum) in yeasts (*Schizosaccharomyces pombe*) involves several proteins (kinases and GTPase) which form a so called septation initiation network (SIN). Plants also contain proteins which are homologous to the yeast SIN proteins which are localized in nucleoli. Remarkably, some of these proteins (such as Arabidopsis SGP1/2 and *Brassica napa* MAP4Ka2) have been shown to complement yeast mutants defective in homologs of these genes, as evidenced by formation of multisepta during their overexpression in *S.pombe*. These data suggest the existence of a plant-specific nucleolar SIN-like network with important roles in the cytokinesis and cell cycle regulation (Champion et al., 2004).

### Pre-mRNA splicing and growth and development

Pre-mRNA splicing is crucial to the regulation of gene expression in eukaryotes. As mentioned above, the nucleolus and particularly CBs play important roles in snRNP synthesis, which are essential for the formation of spliceosomes (reviewed in Love et al., 2017). The EJC and particularly two of its core subunits such as MAGO and Y14, are widely known to have essential multiple developmental roles in animals, whereas information for such roles in plants is more limited (Gong and He, 2014; Yang et al., 2016). It has been shown that MAGO proteins are responsible for male fertility (*Physalis floridana*; He et al., 2007), pollen grain development (Arabidopsis; Park et al., 2009) and spermatogenesis (*Marsilea vestita*; Boothby and Wolniak, 2011). The MAGO and Y14 proteins in rice also appear to be involved in floral and vegetative growth, stamen development and pollen maturation. In addition, one of two rice isoforms of Y14 has been shown to be involved in embryogenesis (Gong and He, 2014). The growth and development of other plant organs are also affected by MAGO and Y14: roots, shoots, seed and root hairs (Chen et al., 2007; Park et al., 2009). Interestingly, Y14 and MAGO have been shown to selectively bind pre-messenger RNA of UNDEVELOPED TAPETUM1 (OsUDT1), which is a key controller of stamen development. Down regulation of MAGO and Y14 leads to abnormalities in the OsUDT1 transcript splicing, suggesting that rice EJC subunits may regulate this process (Gong and He, 2014).

Another nucleolar protein which is essential for plant development is RID1, a DEAH-box RNA helicase. Studies on a root initiation defective1-1 Arabidopsis mutant (*rid1-1*) (Ohtani et al., 2013) have implicated this protein in a certain subset of splicing events which may differentially regulate specific developmental programmes, such as root and leaf morphogenesis and meristem maintenance.

Further research is required to explore if nucleolar localization of RID1, MAGO and Y14, are completely required for their role in plant growth, development and reproduction.

### Telomerase maintenance and plant growth and development

Telomeres, are specific DNA–protein structures located at the ends of eukaryotic chromosomes which contain repetitive nucleotide sequences that protect chromosomes from in appropriate attack by endogenous DNA nucleases. Telomere shortening can lead to chromosomal degradation which can culminate in aging and ultimately cell death. To counteract this, plants and other organisms have evolved strategies to maintain telomere length, which predominantly operates via the activity of telomerase, an RNP-based enzyme which consists of telomerase reverse transcriptase, telomerase RNA (TR), and other associated proteins (reviewed in Procházková Schrumpfová et al., 2016). The catalytic subunits of this complex (TERTs) possess multiple nuclear export/localization signals and have been shown to localize to the nucleus and the nucleolus. Furthermore, a preferential nucleolar accumulation was also shown for telomere binding proteins and the telomerase RNA-binding protein, dyskerin (Dvorácková et al., 2010; Dvoráčková et al., 2015). Finally, telomeres as well as subtelomeric regions (flanking the telomeres) also tend to associate with the nucleolus (Pontvianne et al., 2016). The concentration of various telomere-related components in the plant nucleolus strongly suggests a functional link between this sub-nuclear structure and telomere biology. This suggestion is supported by recent observations showing that in Arabidopsis null mutants for the *NUCLEOLIN 1* gene, which have altered rRNA gene expression and overall nucleolar structure, telomeres were shortened and had reduced association with the nucleolus (Pontvianne et al., 2016). Moreover, it was found that NUCLEOLIN 1 physically interacts with a macromolecular complex possessing telomerase activity. These data strongly implicate the nucleolus (and its protein, nucleolin 1) in plant telomere maintenance.

TERT is developmentally regulated in plants (Procházková Schrumpfová et al., 2016). In Arabidopsis plants, the activity of telomerase is low in vegetative tissues but high in reproductive organs. However, application of exogenous auxin, can overcome this developmental regulation and potentiates telomerase activity in mature leaves (Ren et al., 2007); a phenomenon which may be regulated by the Arabidopsis transcription factor TELOMERASE ACTIVATOR1 (TAC1). It has also been shown that telomerase activity in tobacco suspension cells significantly increases at early S-phase of the cell cycle due to auxin, but interestingly abscisic acid (ABA), a plant hormone which can induce the cyclin-dependent protein kinase inhibitor, readily abolishes this effect. These results suggest that antagonistic functions of ABA and auxin in the cell cycle-dependent modulation of telomerase activity in tobacco may be governed via reciprocal phosphorylation and dephosphorylation of telomerase complexes (Yang et al., 2002). A major future challenge is to elucidate the role of nucleolar environment in the cross-talk between plant telomerase and developmental pathways.

Another observation which may link nucleolar functions and plant growth and developmental pathways is that Arabidopsis TR is able to interact with dyskerin which is known to be a component of nucleoli and CBs (Procházková Schrumpfová et al., 2016).

### Nucleolar miRNAs and development

In plants, small 21–24 nucleotide miRNA molecules play important “roles in post-transcriptional gene regulation by base pairing with their complementary mRNA targets” (Li and Zhang, 2016). Mutations in the genes involved in biogenesis and the regulatory roles of miRNAs produce strong effects on development; implicating miRNAs in a broad range of physiological and developmental processes (Li and Zhang, 2016). Taking into account that the nucleolus is involved in RNA silencing pathways it looks natural that such an involvement may be important for plant growth and development. Moreover, in human cells, several miRNAs are highly and specifically localized in nucleoli relative to other compartments. The presence of miRNAs in the nucleolus is independent of DICER and the RNA polymerase I transcription activity of the nucleolus, however it is dependent on CRM1, which is known to be related to nucleolar trafficking of snoRNAs. These data demonstrate the spatial arrangement and complexity of miRNA regulation (Bai et al., 2014). It is enticing to theorize that there might also be specific variability in nucleolar miRNA content which may be dependent on cell type and physiological state, and which could regulate developmental processes.

### Fibrillarin and systemic macromolecular trafficking in plants

Plants have evolved a specific network interconnected by plasmodesmata (PD), which are cytoplasmic channels between cells that permit local movement of various molecules. In addition, plants can rapidly transfer nutrients, assimilates and macromolecules over longer distances via the phloem, a specific plant transport system composed of enucleated sieve elements and neighboring companion cells. The phloem and PD form a continuous symplastic connection which can link distant plant organs, and likely play a key role in transmitting macromolecules, such proteins and RNAs including siRNAs/miRNAs, as components of integrated signaling pathways which are central to plant development and controlling responses to both biotic and abiotic stresses (reviewed in Lough and Lucas, 2006; Buhtz et al., 2010). However, to govern such signaling pathways, plants have evolved stringent control systems to prevent molecules other than those that perform necessary functions from trafficking throughout the plant by cell-to-cell (PD) and long-distance (phloem companion cell-sieve element junctions) movement. Indeed, the plant transport network is fully permeable only to some low-molecular weight compounds, but specific “transport” proteins are able to increase the permeability of the control systems, which permits entry of larger macromolecules or macromolecular complexes. The most studied example of active plasmodesmatal transport involves the movement of plant viruses, which use designated virus-encoded movement proteins (Lough and Lucas, 2006) to hijack and manipulate the PD to allow viral particles or their transport forms to pass between cells; thus facilitating systemic invasion. Plant proteins such as fibrillarin (discussed in more detail below) which is an abundant nucleolar protein, is also able to facilitate long distance movement and has been implicated in the systemic spread of various plant viruses, such as groundnut rosette virus (GRV), potato leaf roll virus (PLRV), rice stripe virus (RSV) (Kim et al., 2007a; Zheng et al., 2015) and subviral bamboo mosaic virus-associated satellite RNA (satBaMV; Chang et al., 2016). It would be difficult to expect that plant fibrillarin, whose main function is in rRNA processing and modification, has evolved specifically to assist viruses; it is more likely that viruses hijack fibrillarin's role in phloem RNA trafficking. The phloem transport system and RNAs play a critical role in plant survival and together they likely operate as a complex, multifunctional and regulatory long-distance RNA signaling system. Recently in addition to siRNAs and miRNAs, small non-coding RNA molecules of sizes between 30 and 90 bases have been identified and characterized in pumpkin phloem sap. In addition to fragments of rRNAs and tRNAs, the identified RNAs include phloem-specific spliceosomal RNAs, which also have nucleolar steps in their formation (Zhang et al., 2009).

### Effect of mammalian p53 on plant development

p53 is a key mammalian nucleolar tumor suppressor which plays a pivotal role in molecular stress responses, developmental processes and guarding the genome from DNA damage (Boulon et al., 2010). However, p53 has not been found in plants. It is therefore striking that p53 transgenically expressed in Arabidopsis induced early senescence and excessive inflorescence branching (fasciation) (Ma et al., 2016). This effect in plants is presumed to be due to the elevated homologous DNA recombination directed by p53. SUPPRESSOR OF NPR1-1 INDUCIBLE 1 (SNI1) is a negative regulator of plant homologous recombination (operating with RAD51D downstream of SNI1), which is not present in animals. Interestingly, *sni1* mutants have a fasciated phenotype in the presence of p53, whereas *rad51d* mutants are able to fully suppress the p53-induced phenotype; implicating the SNI1-RAD51D signaling pathway as a regulator of p53 (Ma et al., 2016). The underlying molecular mechanisms of how this signaling pathway is activated by p53, and what nucleolar functions are involved remain to be explored.

As indicated in the above sections, nucleolar components are key to many facets of plant development and growth, moreover in recent years they have also been implicated in modulating responses to exogenous biotic and abiotic stresses.

## Virus infections

Considering the diverse functional roles of the nucleolus, it is unsurprising that this structure is a common target of many types of viruses, including plant viruses (Hiscox, 2007; Greco, 2009; Taliansky et al., 2010). Interestingly, the repertoire of viruses that interact with nucleoli includes not only “nuclear viruses” that replicate within the nucleus, but also “cytoplasmic viruses” (containing mainly positive-strand RNA) in which cytoplasm is an exclusive site of their replication. Since the early studies on plant viruses and their association with the nucleolus (Taliansky et al., 2010), a plethora of proteins from RNA- and DNA-containing viruses which can enter the nucleus and target nucleoli have been described. Moreover, several recent studies have expanded beyond these phenomenological observations and have elucidated the molecular mechanisms underpinning various virus-nucleolar associations and their roles in the plant viral life cycle and disease with potential links to a broader range of biological processes including growth and development. This section will therefore focus on functional and mechanistic implications of the virus–nucleolar interactions (Table 2).

### Direct roles of fibrillarin in the life cycle of plant viruses

Due to its integral role in various RNA processing and RNP assembly events, the nucleolus has evolved as an attractive target for many viruses to exploit its functions in production and transport of viral RNPs. Plant viruses can hijack nucleolar proteins for production of viral RNP particles, replication and movement, and to counter antivirus defense.

For example, in the nucleus, the ORF3 long-distance movement protein of GRV (an umbravirus) targets CBs causing their re-organization into multiple CB-like structures which move to and coalesce with the nucleolus. ORF3 subsequently recruits and uses fibrillarin for assembly of cytoplasmic viral RNP complexes able to move long-distance systemically through the phloem (Canetta et al., 2007; Kim et al., 2007a,b). Localization of ORF3 to the nucleolus is essential for successful systemic infection. In another example, the coat protein (CP) and CP read-through protein of PLRV (polerovirus) are targeted to the nucleolus and although the implications of this from an infection perspective are unelucidated, it is known that nucleolar components such as fibrillarin are required for long-distance movement of PLRV and subsequent systemic plant infections (Haupt et al., 2005; Kim et al., 2007a). A different scenario of fibrillarin-dependent long-distance movement has been recently described for satBaMV (satellite RNA). While the helper virus, bamboo mosaic virus (BaMV, potexvirus) utilizes virus-specific transport machinery composed of movement and capsid proteins for invasion, it does not require fibrillarin (Chang et al., 2016). In contrast, for the BaMV satellite virus (satBaMV) to establish long-distance movement, satBaMV-encoded p20 protein is required to interact with fibrillarin and form RNP complexes with satBaMV, which are competent for trafficking in the phloem.

Plant viruses usually encode silencing suppressor proteins which can counteract RNA-silencing-based defense mechanisms induced by infection. Protein p2 of RSV (tenuivirus) is a weak silencing suppressor and is able to interact with fibrillarin within the nucleolus. Furthermore, fibrillarin depletion (using RNAi knock down) abolished the systemic movement of RSV, suggesting that interaction of fibrillarin with the p2 silencing suppressor facilitates the long-distance RSV movement (Zheng et al., 2015). However in studies of some other viruses, it has been shown that interaction of fibrillarin with viral silencing suppressors modulates virus functions other than the long-distance movement. Indeed, the VPg/NIa silencing suppressor protein of potato virus A (PVA, potyvirus) has activity which depends on VPg localization to CBs and the nucleolus. The NIa interacts with fibrillarin in the nucleolus and CBs via its VPg domain, and depletion (RNAi knock down) of fibrillarin lowers PVA accumulation, but does not compromise the process of virus long-distance movement *per se* (Rajamäki and Valkonen, 2009). These data raise the question as to whether VPg/NIa targets components of RNA silencing pathways that are localized in the nucleolus and CBs (Pontes and Pikaard, 2008) to inhibit silencing machinery and consequently enhance infection.

The poa semilatent virus (PSLV, hordeivirus) movement protein which is encoded by the first gene of the triple gene block (TGBp1) has also been shown to be nucleolar localized and interact with fibrillarin (Semashko et al., 2012). Similar interactions have been observed between fibrillarin and TGB1 encoded by another hordeivirus, *barley stripe mosaic* virus (BSMV), which were found to facilitate cell-to cell movement of the virus (Li et al., 2017).

Collectively these data demonstrate how diverse taxonomic virus groups and functional categories of viral proteins can interact with fibrillarin, to control the disease process. Thus fibrillarin may have new unrecognized activities which are exploited by plant viruses, but which may also be involved in other biological processes (such as growth and development) in healthy plants.

### Role of ribosomal proteins

A growing body of evidence demonstrates that the ribosomal proteins (r-proteins) are not only scaffolds required to maintain the structural integrity of mature ribosomes, but rather, some of them are involved in regulatory activities in various cell cycle, cell death and developmental processes (e.g., Lindström, 2009). In addition, the accumulation of different sets of r-proteins has been shown to be enhanced by many viruses, including various plant viruses (Dardick, 2007; Yang et al., 2007, 2009; Rajamäki et al., 2017). Moreover, some r-proteins have been found to interact with plant viral proteins implicating them as important components of plant-virus interactions.

Of all the r-proteins involved in interactions with viruses RPS6 is the most studied. RPS6 is partially regulated by ribosomal protein S6 kinase (S6K) which, in turn, is a downstream component of TOR signaling pathway and a key modulator of plant responses to stresses and developmental stimuli (Xiong Sheen and Sheen, 2014; Son et al., 2016). In addition to its well-known role as a structural component of the 40S ribosomal subunit, RPS6 is also involved in regulation of rDNA transcription via nucleolar interactions with histone deacetylase 2b (HD2B) and nucleosome assembly protein 1 (NAP1), which is a histone chaperone (Kim et al., 2014; Son et al., 2015). The RPS6-HD2b complex functions as a negative regulator of rRNA synthesis via its binding to and blocking rDNA promoter sites, but this negative effect may be de-repressed by NAP1. Interestingly, S6K-mediated phosphorylation of RPS6 may also activate rDNA transcription presumably causing dissociation of the rDNA transcriptional repression complex.

With regards to virus infection it has been shown that silencing of the *RPS6* and *S6K* genes in *N. benthamiana* decreased accumulation of CMV, PVA and *turnip mosaic* virus (TuMV, potyvirus), which is in contrast to turnip crinkle virus and tobacco mosaic virus (TuCV and TMV respectively; tobamoviruses). This suggests differential requirements for RPS6 and S6K for different virus groups (Rajamäki et al., 2017). While the underpinning mechanisms are still to be elucidated, these observations indicate that there might be interplay between RPS6/S6K activities and plant virus infections. A possible activity of these proteins may be to directly interact with viral proteins and this has recently been shown for potyviral VPg, which is able to form a complex with S6K in the nucleus, nucleolus and cytoplasm. This suggests that potyviruses may recruit S6K to modulate downstream proteins (RPS6 in particular) for enhancing viral invasion. For example, in the nucleolus such an interaction may lead to stimulation of rDNA transcription whereas cytoplasmic interaction may facilitate protein translation. Additional studies are still necessary to explore the role of the nucleolar activities of RPS6 and S6K in relation to virus infections, which may also have implications for other stress responses, growth and development.

### Nucleolar sequestration, storage, and compartmentalization of virus proteins

The nucleolus was demonstrated to be a region of molecule sequestration which may also have activities outside this organelle, such as in the nucleoplasm or cytoplasm (Sirri et al., 2008). The regulation of various nucleolar functions is controlled by the compartmentalization or effusion of specific proteins; a mechanism which may also be hijacked by viruses for localization and storage of viral proteins. For example, cucumber mosaic virus (CMV, cucumovirus) 2b protein is a silencing suppressor that is involved in virus accumulation and virulence (Du et al., 2014), and it is found distributed between the nucleus/nucleolus and the cytoplasm. In the nucleolus this protein interacts with the Argonaute 4 silencing machinery (González et al., 2010), however, neither of these interactions nor nucleolar localization are sufficient for suppression of RNA silencing (González et al., 2012). Instead, it has been shown that it is the cytoplasmic portion of 2b that predominantly possess silencing suppressor activity. It was found that enhanced nuclear and nucleolar 2b accumulation increases virulence and accelerates symptom production, which is independent of its effect on RNA silencing. Thus, it has been suggested that nuclear/nucleolar and cytoplasmic partitioning of the 2b protein between these compartments permit CMV to regulate the equilibrium between damage to the plant and virus accumulation, presumably to optimize virus accumulation (Du et al., 2014).

Alfalfa mosaic virus (AlMV, alfamovirus) CP is a multifunctional protein required not only for virion assembly but also for translation, cell-to-cell and systemic movement (Bol, 2005). The AlMV CP localizes in both the nucleus/nucleolus and cytoplasm. The data shows that the nucleolar import signal masks the RNA-binding activities of AlMV CP, which are required for viral translation and replication; this suggests a model in which the virus life cycle is precisely regulated by the balance between the cytoplasmic/nuclear localization of the CP (Herranz et al., 2012).

In more recent work (Aparicio and Pallás, 2017), AlMV CP has been shown to interact with transcription factor (TF) ILR3, a basic helix–loop–helix family member of TFs, which were suggested to operate in a number of metabolic pathways (Toledo-Ortiz et al., 2003). ILR3 can regulate NEET in Arabidopsis, a key protein in plant senescence, development, reactive oxygen species (ROS) modulation and iron metabolism (Nechushtai et al., 2012). The AlMV CP–ILR3 interaction causes partial re-distribution of this TF from the nucleus to the nucleolus and this re-distribution may cause down-regulation of NEET, which can induce plant hormone responses, which may form a hormonal balance optimal for plant viability and virus production (Aparicio and Pallás, 2017).

Another viral protein which is sequestered into the nucleus to regulate its activity is the P19 silencing suppressor encoded by the tomato bushy stunt virus (TBSV, tombusvirus). For example, P19 silencing suppressor activity is realized in the cytoplasm, however host plant ALY proteins (mRNA-processing-export factors) can potentially interfere with this function by redistributing P19 to the nucleus/nucleolus, where it cannot reach its target silencing RNA (Canto et al., 2006); this constitutes a novel plant defense mechanism which blocks silencing suppression.

Taken together, these data show that various plant host nucleolar proteins, in addition to their traditional roles, may have other diverse natural functions which are widely exploited by viruses for their own benefits. Interestingly, some of these proteins as mentioned above are also involved in plant perception and responses to environmental and developmental cues. Further detailed investigation of molecular mechanisms underlying the virus-nucleolus interactions will provide new insights into our understanding of intriguing multifunctional complexity of the nucleolus including its role in plant growth and development.

## Pathogens other than viruses

Several lines of evidence demonstrate that plant pathogens other than viruses, also target the nucleolus (Table 1). In particular, some effector proteins expressed by plant pathogens which aid infection and favor parasitism, have been shown to localize to nucleoli (Chaudhari et al., 2014). For example, *Globodera pallida*, a potato cyst nematode, delivers two protein effectors encoded by gene members of the SPRYSEC family (*22E10* and *13G11*), into the nucleolus presumably to suppress host defense (Jones et al., 2009). Nucleolar localization has also been demonstrated for several effectors encoded by filamentous pathogens (oomycetes and fungi), such as the poplar leaf rust fungus *Melampsora larici-populina* (Petre et al., 2015) and the broad-host-range oomycete *Phytophtora capsici* (Stam et al., 2013). Interestingly, a plant E3 ligase (CMPG1) which is involved in host resistance against *P. infestans*, also accumulates in the nucleolus when stabilized by the Avr3a effector protein (Gilroy et al., 2011). These data, although merely descriptive, suggests that the nucleolus may be an important controller of host defense mechanisms against a broad range of pathogens.

This hypothesis is supported by more functional and mechanistic studies carried out using *Hyaloperonospora arabidopsidis* and *P. infestans*. Several RXLR effectors encoded by the obligate biotrophic oomycete pathogen *Hyaloperonospora arabidopsidis* (the causal agent of downy mildew), have been shown to localize to the nucleolus of plant cells (Leonelli et al., 2011; Caillaud et al., 2012) and regulate plant responses. For example, the effector HaRxL44 interacts with nuclear and nucleolar Mediator subunit 19a (MED19a), a component of the Mediator complex involved in the association between RNA polymerase II and transcriptional regulators. This interaction leads to MED19a degradation in a proteasome-dependent manner, which switches transcription of plant defense genes from the salicylic acid-responsive pathway to the jasmonic acid and ethylene-responsive pathways; demonstrating that this nucleolar pathogen effector alters host transcription to enhance susceptibility to infection (Caillaud et al., 2013). In contrast, other nucleolar *H. arabidopsidis* effectors, such as ATR13 Emco5, may interact with host RPP13-Nd (a cognate R-gene product) which triggers programmed cell death and limits pathogen spread (Leonelli et al., 2011).

The *P. infestans* RXLR effector Pi04314 enhances leaf colonization through its nuclear activity which can regulate activation of salicylic and jasmonic acid-responsive defense pathways. Pi04314 associates with three isoforms of the nucleolar host protein phosphatase 1 catalytic (PP1c) unit, inducing their re-distribution from the nucleolus to the nucleoplasm. A model has been proposed whereby Pi04314 interacts with PP1c isoforms to form holoenzymes, which attenuate transcriptional responses of host plant defense genes to promote late blight disease (Boevink et al., 2016).

In light of these findings it could be proposed that plant pathogens deliver effectors to alter host processes *via* activities in the host nucleolus. Understanding of how effectors target and manipulate host proteins and elucidating the function of the nucleolus in these processes is a critical area which needs further exploration.

## The plant nucleolus under stress

In mammalian cells, various types of stress often affect the nucleolus by inducing complex and diverse changes in its size, structure and protein composition. Proteomic analysis has revealed a broad network comprising different nucleolar proteins involved in stress responses (Boulon et al., 2010) and suggested that the mammalian nucleolus can function as a key regulator in stress sensing, perception and response. Although in plants, cross-talk between nucleolar functions and stress signaling pathways has been less well studied, there are multiple sources of evidence which suggests that the plant nucleolus also has a direct role in sensing stresses such as drought, salinity and inclement temperature and responding by modulating a variety of different pathways, which may increase stress tolerance.

It has been shown that stress can be accompanied by dramatic morphological alterations in the protein content and organization of plant nucleoli. These changes are presumably related to alterations in diverse nucleolar transcriptional activity under stress conditions. For example, in soybean meristemic root cells exposed to low temperature stress, transcriptional activity is reduced and this is reflected in the looser structure and size increase of nucleoli, but decrease in the number of FCs and DFCs (Stepinski, 2009). In this study decreases in the amounts of important nucleolar proteins (fibrillarin and B23) were also observed.

As mentioned above, in mammalian cells, one of the major regulators of cellular responses to diverse stresses including genome damage (DNA damage response, DDR) is the p53 transcription factor (Boulon et al., 2010). DDR is a key process to maintain genome stability and protect DNA from damage caused by numerous endogenous and exogenous DNA damaging agents. It has been found that disintegration of the nucleolus, by drugs or UV irradiation can mediate activation of p53 pathways, and suggested that the nucleolus itself can act as an upstream stress sensor. p53 has not been found in plants indicating that plants may possess their own, unique system(s) for stress responses and genome stability maintenance in particular, and recent evidence has implicated several plant nucleolar proteins in such responses (Table 1, Figure 2).

Plant SUPPRESSOR OF GAMMA RESPONSE 1 (SOG1) operates as a transcription factor and master regulator of DDR that has many similarities with p53. Like p53, SOG1 activates transcription of more than 100 genes that control cell cycle, DNA repair and programmed cell death (Yoshiyama et al., 2014; Yoshiyama, 2016). The *sog1-1* mutant exhibits increased resistance of root growth to zeocin and no cell cycle arrest and PCD in response to DNA double-strand breaks (DSB) (Yoshiyama, 2016).

There are many other plant proteins that are involved in plant DDR which have been extensively reviewed by Manova and Gruszka (2015), Yoshiyama et al. (2014), Yoshiyama (2016), and Donà and Mittelsten Scheid (2015). Among them are TDP1, RECQ4A, and RTEL1. Like its human or yeast homologs, Arabidopsis tyrosyl-DNA phosphodiesterase 1 (TDP1) for example, is able to repair DNA damage in which topoisomerases can occasionally covalently bind the ends of DNA strand breaks, by hydrolyzing the 3′-phosphotyrosyl bond between topoisomerase and DNA (Lee et al., 2010). Interestingly, inability of the Arabidopsis *tdp* mutants to repair DNA damage results in plant dwarfing and extensive cell death during development (Lee et al., 2010). This is consistent with later studies which show that RNAi silencing of the *TDP1* gene in *Medicago truncatula* results in significant changes in gene expression, which is manifested in reduced cell division, perturbed plant growth and early leaf senescence (Donà et al., 2013). *TDP1* knockdown is known to impinge on rRNA processing and ribosome biogenesis and disrupts the structure and architecture of the nucleolus, as revealed by electron microscopic analysis (Donà et al., 2013). It is also worth noting that deficiency of the TDP1 protein leads to telomere shortening (Donà et al., 2013). Taken together these findings strongly implicate the nucleolus as a central component of TDP-dependent DDR and developmental processes.

Arabidopsis REQ4A helicase is involved in dissolution of Holliday junctions, aberrant DNA structures that are formed during DNA replication and recombination, by suppressing homologous recombination and producing non-crossover recombinants (Hartung et al., 2007). However, to achieve this activity RECQ4A usually acts as a part of the RTR protein complex also containing topoisomerase TOP3α and structural proteins RMI1 and RMI2. Interestingly, another Arabidopsis protein, Regulator of Telomere ELongation helicase 1 (RTEL1), which is normally involved in telomere maintenance is also able to reduce efficiency of homologous recombination (Röhrig et al., 2016). Moreover, both RMI2 and RTEL1 have recently been shown to safeguard stability of 45S rDNA repeats. Plants defective in both these proteins exhibit male infertility, implicating functional links between suppression of homologous recombination and plant developmental defects especially in highly proliferative tissues such as the male germline, where reduction of 45S rDNA repeats seems to be most pronounced (Röhrig et al., 2016). Intriguingly, plant REQ4B helicase which is closely related to RECQ4A, has an antagonistic function and promotes formation of crossovers; exhibiting a role which has not been recognized for any other eukaryotic RECQ-like helicases (Hartung et al., 2007).

Although some DDR and DNA repair factors (such as TDP1) are associated with nucleoli, the precise role of the nucleolus in DDR remains to be elucidated.

DEAD-box RNA helicases, STRESS RESPONSE SUPPRESSOR1 (STRS1) and STRS2 proteins function as negative regulators of stress-induced gene expression. They are typically active in unstressed plants and serve to reduce high level constitutive expression of stress-responsive genes which may be detrimental to plant growth and development (Khan et al., 2014). Under normal conditions, the STRS proteins are mainly localized in the nucleolus and chromocentres, which is suggestive of their site of function. However, in response to salt or heat stresses, the STRS proteins exhibit rapid relocalization into nucleoplasm, presumably activating stress responses. STRS defective mutants (*strs*) exhibit enhanced tolerance to salt, osmotic and heat stress whereas *STRS* overexpression leads to diminished tolerance (Khan et al., 2014). Interestingly, in Arabidopsis mutants that are defective in RNA-mediated gene silencing, such as the RNA-directed DNA methylation (RdDM) pathway, the STRS proteins have been shown to mis-localize. Furthermore, it has been found that heterochromatic RdDM target loci have enhanced expression and lowered DNA methylation in the *strs* mutants, which indicates that the STRS proteins could participate in the epigenetic silencing of stress-responsive gene expression (Khan et al., 2014).

Another protein which is redistributed in response to stress conditions (for example, hypoxia) is eIF4A-III, one of the core EJC components (Koroleva et al., 2009). However, in contrast to STRSs, eIF4-III has been shown to concentrate in the nucleolus and splicing speckles under this kind of stress. It is possible that in such hypoxia conditions, the eIF4A-III may retain certain mRNAs in the nucleolus, which could prevent their movement to the cytosol and subsequent translation.

Several other plant proteins involved in stress responses have also been relocalized to the nucleolus and have been implicated in growth and development. For example the Rab 28 Late Embryogenesis Abundant (LEA) protein has been observed to accumulate in nucleoli, and transgenic overexpressors of this protein exhibit higher relative water content, increased root and leaf areas, and reduced chlorophyll loss compared with wild-type plants when grown under osmotic stress (Amara et al., 2013). As an additional example, AtREN1 protein (an early male gametophytic gene, At1g77570, which is strongly homologous to the heat shock transcription factor gene *HSFA5*) has been shown to accumulate in the nucleolus, and plants mutated in this gene have structural and functional abnormalities in male gametophyte development, pollen grain development and perturbed heat stress responses relative to wild-type plants (Renák et al., 2014). Taken together this is indicative of a complex integrated signaling mechanism which links nucleolar functions, pollen development and heat stress in Arabidopsis.

## Functional association of the nucleolus, cajal bodies and poly(ADP-ribose) polymerase

In physical and functional coordination with nucleoli, CBs play many important roles in RNA metabolism and formation of RNPs involved in transcription, splicing, ribosome biogenesis, and telomere maintenance (reviewed by Love et al., 2017). In addition, like the nucleolus, plant CBs participate in various other non-canonical functions, such as modulating plant responses to virus infections and abiotic stresses (Shaw et al., 2014). For example it was previously observed that coilin (the hallmark protein of CBs) could differentially affect the interactions of plants with viruses of diverse taxa. In this study it was found that coilin deficiency and/or CB depletion could increase accumulation and systemic infection by some viruses like *barley stripe mosaic* virus (BSMV, hordeivirus), tobacco rattle virus (TRV, tobravirus), tomato black ring virus (TBRV, nepovirus) and tomato golden mosaic virus (TGMV, begomovirus), while for other viruses such as potato virus Y (PVY, potyvirus) and turnip vein clearing virus (TVCV; tobamovirus) the opposite phenomenon was observed (Shaw et al., 2014). These data clearly show that coilin/CBs are important in regulating virus pathogenesis in plants. Coilin gene suppression in plants could also confer salt tolerance (Love et al., 2017), which *in toto* implicates CBs in plant perception and responses to stress.

While these underlying processes remain poorly elucidated, a possible mechanism may lie within recent studies which show an intimate association between the nucleolus, CBs and the poly (ADP-ribose) polymerase (PARP) family member, PARP1. In dissected *Drosophila* salivary gland cells, coilin and fibrillarin are known to interact with PARP1 (Kotova et al., 2009), a nuclear protein which has important regulatory functions in DNA repair and genotoxic stress tolerance, transcription, cell cycle control and programmed cell death (PCD) (Kotova et al., 2009; Briggs and Bent, 2011; Luo and Kraus, 2012; Ji and Tulin, 2013; Schulz et al., 2014). PARP1 modifies the function of a variety of nuclear “target” proteins by attaching chains of ADP ribose (PAR) to them and itself. Although most of the PARP1 molecules bind chromatin and accumulate in the nucleolus, automodified PARP1 has been shown to interact non-covalently via PAR polymers with some nucleolar and CB components, including fibrillarin and coilin respectively (Kotova et al., 2009). These associations may mediate the shuttling of PARP1 and PAR-containing protein complexes from the nucleolus and chromatin into CBs. This movement is presumed to be required for PAR removal and recycling, which may act as a molecular switch which modulates the functional activities of PARP1.

In plants, such activities are thought to be involved in the regulation of several physiological processes, including responses to abiotic and biotic stresses, differentiation and cell cycle control (reviewed in Briggs and Bent, 2011; Love et al., 2017). For example, it has been shown that PARP deficiency in Arabidopsis and *Brassica napus* (oilseed rape) plants leads to increased tolerance to drought and heat stress (De Block et al., 2005). On the other hand, overexpression of *PARP2* in Arabidopsis decreased the number of DNA nicks at high concentrations of H_2_O_2_ but increased their number at low H_2_O_2_ concentrations (reviewed in Briggs and Bent, 2011). In addition, PARP inhibitors reduce damage to tobacco and soybean cells from oxidative stress and heat shock (Amor et al., 1998; Tian et al., 2000). PARP also markedly affects plant-pathogen interactions. In particular, PARP knockout mutants of Arabidopsis have been found to display increased susceptibility to *Pseudomonas syringae*, indicating that PARP is required for antibacterial resistance. In agreement with these observations, PARP inhibitors have been shown to block some basal plant defense responses such as cell wall reinforcement with callose and lignin, which are induced by microbe-associated molecular patterns, such as bacterial flagellin or EF-Tu epitopes (Adams-Phillips et al., 2010). It has been shown that callose deposition provides a physical barrier that blocks spread of virus infection through the plasmodesmata (Li et al., 2012), and it would be interesting to speculate whether PARP may be involved in this process. While the evidence indicates that PARP is a central factor which controls resistance to various plant pathogens, plant PARP-mediated activities have also been implicated in differentiation and cell cycle control pathways, which are known to overlap with components of plant stress signaling pathways (reviewed in Briggs and Bent, 2011). Alterations in the poly(ADP-ribosyl)ation level induced by extrinsic (biotic or environmental) or intrinsic (genetic/physiological) cues play an important role in plant stress signaling and developmental processes (Briggs and Bent, 2011). It is possible that in such cases, the PARP levels could be controlled via coilin and fibrillarin induced trafficking and redistribution of automodified PARP and other PARylated proteins from the nucleolus to CBs for recycling. Although this mechanism may transduce responses to developmental and stress cues, it is also an intriguing possibility that they may underpin at least some of the architectural and protein content changes in CBs and the nucleolus. Future work will be required to elucidate these possibilities further.

## Perspectives

The nucleolus is involved in coordinating many major biological processes such as ribosome production, spliceosome formation, gene expression regulation (e.g., transcriptional/post-transcriptional gene silencing), mRNA surveillance and telomere maintenance (Figure 2). It is therefore unsurprising that this prominent sub-nuclear domain has been repeatedly implicated as an important regulator of signaling pathways which control plant growth and development, disease and stress responses (Tables 1, 2, Figure 2). This is particularly intriguing especially considering that in the last decade it has been found that there is frequent cross-talk between components or facets of these pathways, which may in turn regulate or be regulated by the nucleolus and associated CBs. In spite of this we are still far from comprehensively understanding the molecular mechanisms underpinning such control systems, and much remains uncharacterized.

For instance, it is becoming particularly interesting to explore if plant cells can produce more than one type of ribosome. What are the external (stress and/or disease) or internal (developmental) factors that may cause modification of RPs or rRNAs, giving rise to formation of distinct “specialized” (“renegade”; Lafontaine, 2015) ribosomes? Can such ribosome reprogramming be tissue- or organ-specific and differentially affect translation in response to stress or disease to provide mechanisms underpinning developmental regulation and biotic and environmental stress defense?

An increasing number of viral proteins and proteins encoded by other pathogens (effectors) have been shown to target nucleoli and CBs of infected plants. What are the mechanisms of such targeting and what are the molecular consequences of this targeting with respect to the host defense response?

This review has provided various examples demonstrating that some viruses have evolved to be able to exploit some nucleolar proteins (fibrillarin in particular), for their own benefits (e.g., virus movement throughout the infected plant). It would be especially intriguing to elucidate the molecular mechanisms of this phenomenon, given the essential role of fibrillarin in absolutely different process of modification and processing of rRNA. Is fibrillarin also involved in other processes of macromolecular trafficking in plants or interaction with other pathogens, e.g., controlling or assisting transport of regulatory or signaling mRNAs or ncRNAs in plants? What are the other nucleolar proteins interacting with pathogen effectors?

In future, uncovering the large-scale protein-protein interactome networks will be required to elucidate the contextual mechanisms and molecular switches which underpin nucleolar activities and physiological control. Such work will identify key nucleolar regulators of different plant signaling responses, which shall provide real targets for crop improvement by allowing us to tailor how plants respond to particular forms of environmental stress.

## Author contributions

MT and NK contributed to the review conception, literature analysis and writing. AL contributed to the review editing, writing and data evaluation. SM and AM were involved in writing the Chapter “Virus Infection” and designing Figures and Table.

### Conflict of interest statement

The authors declare that the research was conducted in the absence of any commercial or financial relationships that could be construed as a potential conflict of interest.

## Abbreviations

rRNA: ribosomal RNA
RNP: ribonucleoprotein
FC: fibrillar centre
DFC: the dense fibrillar component
GC: the granular component
snoRNA: small nucleolar RNA
snRNAs: small nuclear RNAs
NoV: nucleolar vacuole
NAD: nucleolus-associated chromatin domain
TE: transposable element
NOR: nucleolus organizer region
Pol II-RNA: polymerase II
Pol I: RNA polymerase I
snoRNP: small nucleolar ribonucleoprotein
EJC: Exon junction complex
NMD: nonsense-mediated mRNA decay
scaRNAs: small Cajal bodies-specific RNAs
CB: Cajal body
siRNA: small interfering RNA
ncRNA: non-coding RNA
sRNA: small RNA
ta-siRNA: trans-acting small interfering RNA
natsiRNA: natural cis-antisense siRNA
miRNA: microRNA
hc-siRNA: heterochromatic small interfering RNA
TGS and PTGS: transcriptional and post transcriptional gene silencing
DCL: DICER-like enzyme
RDR: RNA-dependent RNA polymerase
TR: telomerase RNA
TERT: telomerase reverse transcriptase
RBFs: ribosome biogenesis factors
TOR: target of rapamycin
SIN: septation initiation network
TAC1: telomerase activator 1
ABA: abscisic acid
GRV: groundnut rosette virus
PLRV: potato leaf roll virus
RSM: rice stripe virus
BaMV: bamboo mosaic virus
satBaMV: satellite RNA associated with bamboo mosaic virus
SNI1: suppressor of NPR1-1inducible 1
CP: coat protein
PVA: potato virus A
TGBp1: protein 1 encoded with triple gene block
PSLV: poa semilatent virus
S6K: S6 kinase
RPS6: ribosomal protein S6
HD2B: histone deacetylase 2
NAP1: nucleosome assembly protein 1
TuMV: *turnip mosaic* virus
TuCV: turnip crinkle virus
TMV: tobacco mosaic virus
CMV: cucumber mosaic virus
AlMV: Alfalfa mosaic virus
TF: transcription factor
TBSV: tomato bushy stunt virus
STRS: stress response suppressor
RdDM: RNA-directed DNA methylation
LEA: late embryogenesis abundant protein
TRV: tobacco rattle virus
BSMV: *barley stripe mosaic* virus
TBRV: tomato black ring virus
TGMV: tomato golden mosaic virus
TVCV: turnip vein clearing virus
PVY: potato virus Y
PARP: poly(ADP ribose)polymerase
PAR: ADP-ribose polymer
PCD: programmed cell death
SOG: suppressor of gamma response
DDR: DNA damage response
DSB: double-strand breaks
TDP: tyrosyl-DNA phosphodiesterase
RTEL: regulator of telomere elongation helicase.

## References

[B1] Adams-Phillips L. Briggs A. G. Bent A. F. (2010). Disruption of poly(ADP-ribosyl)ation mechanisms alters responses of Arabidopsis to biotic stress. Plant Physiol. 152, 267–280. 10.1104/pp.109.148049 19889874 PMC2799362

[B2] Ahmad Y. Boisvert F. M. Gregor P. Cobley A. Lamond A. I. (2009). NOPdb: nucleolar Proteome Database−2008 update. Nucleic Acids Res. 37, 181–184. 10.1093/nar/gkn804 18984612 PMC2686505

[B3] Amara I. Capellades M. Ludevid M. D. Pagès M. Goday A. (2013). Enhanced water stress tolerance of transgenic maize plants over-expressing LEA Rab28 gene. J. Plant Physiol. 170, 864–873. 10.1016/j.jplph.2013.01.004 23384757

[B4] Amor Y. Babiychuk E. Inzé D. Levine A. (1998). The involvement of poly(ADP-ribose) polymerase in the oxidative stress responses in plants. FEBS Lett. 440, 1–7. 10.1016/S0014-5793(98)01408-2 9862413

[B5] Andersen J. S. Lyon C. E. Fox A. H. Leung A. K. Lam Y. W. Steen H.. (2002). Directed proteomic analysis of the human nucleolus. Curr. Biol. 12, 1–11. 10.1016/S0960-9822(01)00650-9 11790298

[B6] Aparicio F. Pallás V. (2017). The coat protein of Alfalfa mosaic virus interacts and interferes with the transcriptional activity of the bHLH transcription factor ILR3 promoting salicylic acid-dependent defence signalling response. Mol. Plant Pathol. 18, 173–186. 10.1111/mpp.12388 26929142 PMC6638206

[B7] Bai B. Liu H. Laiho M. (2014). Small RNA expression and deep sequencing analyses of the nucleolus reveal the presence of nucleolus-associated microRNAs. FEBS Open Bio. 4, 441–449. 10.1016/j.fob.2014.04.010 24918059 PMC4050192

[B8] Bazeley P. S. Shepelev V. Talebizadeh Z. Butler M. G. Fedorova L. Filatov V.. (2008). snoTARGET shows that human orphan snoRNA targets locate close to alternative splice junctions. Gene 408, 172–179. 10.1016/j.gene.2007.10.037 18160232 PMC6800007

[B9] Beven A. F. Lee R. Razaz M. Leader D. J. Brown J. W. Shaw P. J. (1996). The organization of ribosomal RNA processing correlates with the distribution of nucleolar snRNAs. J. Cell Sci. 109, 1241–1251. 8799814 10.1242/jcs.109.6.1241

[B10] Boevink P. C. Wang X. McLellan H. He Q. Naqvi S. Armstrong M. R.. (2016). A Phytophthora infestans RXLR effector targets plant PP1c isoforms that promote late blight disease. Nat. Commun. 7:10311. 10.1038/ncomms10311 26822079 PMC4740116

[B11] Boisvert F. M. van Koningsbruggen S. Navascués J. Lamond A. I. (2007). The multifunctional nucleolus. Nat. Rev. Mol. Cell Biol. 8, 574–585. 10.1038/nrm2184 17519961

[B12] Bol J. F. (2005). Replication of alfamo- and ilarviruses: role of the coat protein. Annu. Rev. Phytopathol. 43, 39–62. 10.1146/annurev.phyto.43.101804.120505 16078876

[B13] Boothby T. C. Wolniak S. M. (2011). Masked mRNA is stored with aggregated nuclear speckles and its asymmetric redistribution requires a homolog of Mago nashi. BMC Cell Biol. 12:45. 10.1186/1471-2121-12-45 21995518 PMC3205038

[B14] Bos J. I. Armstrong M. R. Gilroy E. M. Boevink P. C. Hein I. Taylor R. M.. (2010). Phytophthora infestans effector AVR3a is essential for virulence and manipulates plant immunity by stabilizing host E3 ligase CMPG1. Proc. Natl. Acad. Sci. U.S.A. 107, 9909–9914. 10.1073/pnas.0914408107 20457921 PMC2906857

[B15] Boulon S. Westman B. J. Hutten S. Boisvert F. M. Lamond A. I. (2010). The nucleolus under stress. Mol. Cell. 40, 216–227. 10.1016/j.molcel.2010.09.024 20965417 PMC2987465

[B16] Bouvet P. Diaz J. J. Kindbeiter K. Madjar J. J. Amalric F. (1998). Nucleolin interacts with several ribosomal proteins through its RGG domain. J. Biol. Chem. 273, 19025–19029. 10.1074/jbc.273.30.19025 9668083

[B17] Briggs A. G. Bent A. F. (2011). Poly(ADP-ribosyl)ation in plants. Trends Plant Sci. 16, 372–380. 10.1016/j.tplants.2011.03.008 21482174

[B18] Brighenti E. Treré D. Derenzini M. (2015). Targeted cancer therapy with ribosome biogenesis inhibitors: a real possibility? Oncotarget 6, 38617–38627. 10.18632/oncotarget.5775 26415219 PMC4770724

[B19] Brown J. W. Shaw P. J. (1998). Small nucleolar RNAs and pre-rRNA processing in plants. Plant Cell. 10, 649–657. 10.1105/tpc.10.5.649 9596627 PMC1464647

[B20] Brown J. W. Shaw P. J. (2008). The role of the plant nucleolus in pre-mRNA processing. Curr. Top. Microbiol. Immunol. 326, 291–311. 10.1007/978-3-540-76776-3_16 18630759 PMC7121088

[B21] Buhtz A. Pieritz J. Springer F. Kehr J. (2010). Phloem small RNAs, nutrient stress responses, and systemic mobility. BMC Plant Biol. 13:64 10.1186/1471-2229-10-64 PMC2923538 20388194

[B22] Caillaud M. C. Asai S. Rallapalli G. Piquerez S. Fabro G. Jones J. D. (2013). A downy mildew effector attenuates salicylic acid-triggered immunity in Arabidopsis by interacting with the host mediator complex. PLoS Biol. 11:1001732. 10.1371/journal.pbio.1001732 24339748 PMC3858237

[B23] Caillaud M. C. Wirthmueller L. Fabro G. Piquerez S. J. Asai S. Ishaque N.. (2012). Mechanisms of nuclear suppression of host immunity by effectors from the Arabidopsis downy mildew pathogen Hyaloperonospora arabidopsidis (Hpa). Cold Spring Harb. Symp. Quant. Biol. 77, 285–293. 10.1101/sqb.2012.77.015115 23211925

[B24] Canetta E. Kim S. H. Kalinina N. O. Shaw J. Adya A. K. Gillespie T.. (2007). A plant virus movement protein forms ringlike complexes with the major nucleolar protein, fibrillarin, *in vitro*. J. Mol. Biol. 376, 932–937. 10.1016/j.jmb.2007.12.039 18199452 PMC7126915

[B25] Canto T. Uhrig J. F. Swanson M. Wright K. M. MacFarlane S. A. (2006). Translocation of Tomato bushy stunt virus P19 protein into the nucleus by ALY proteins compromises its silencing suppressor activity. J. Virol. 80, 9064–9072. 10.1128/JVI.00953-06 16940518 PMC1563904

[B26] Carron C. Balor S. Delavoie F. Plisson-Chastang C. Faubladier M. Gleizes P. E.. (2012). Post-mitotic dynamics of pre-nucleolar bodies is driven by pre-rRNA processing. J. Cell Sci. 125, 4532–4542. 10.1242/jcs.106419. 22767511

[B27] Champion A. Jouannic S. Guillon S. Mockaitis K. Krapp A. Picaud A.. (2004). AtSGP1, AtSGP2 and MAP4K alpha are nucleolar plant proteins that can complement fission yeast mutants lacking a functional SIN pathway. J. Cell Sci. 117, 4265–4275. 10.1242/jcs.01200 15292395

[B28] Chandrasekhara C. Mohannath G. Blevins T. Pontvianne F. Pikaard C. S. (2016). Chromosome-specific NOR inactivation explains selective rRNA gene silencing and dosage control in Arabidopsis. Genes Dev. 30, 177–190. 10.1101/gad.273755.115 26744421 PMC4719308

[B29] Chang C. H. Hsu F. C. Lee S. C. Lo Y. S. Wang J. D. Shaw J.. (2016). The Nucleolar Fibrillarin protein is required for helper virus-independent long-distance trafficking of a subviral satellite RNA in plants. Plant Cell. 28, 2586–2602. 10.1105/tpc.16.00071 27702772 PMC5134973

[B30] Chaudhari P. Ahmed B. Joly D. L. Germain H. (2014). Effector biology during biotrophic invasion of plant cells. Virulence 5, 703–709. 10.4161/viru.29652 25513771 PMC4189876

[B31] Chen Y. J. C. Wang H. J. Jauh G. Y. (2016). Dual Role of a SAS10/C1D Family Protein in Ribosomal RNA Gene Expression and Processing Is Essential for Reproduction in *Arabidopsis thaliana*. PLoS Genet. 12:e1006408. 10.1371/journal.pgen.1006408 27792779 PMC5085252

[B32] Chen Y. R. Shaw J. F. Chung M. C. Chu F. H. (2007). Molecular identification and characterization of Tcmago and TcY14 in Taiwania (*Taiwania cryptomerioides*). Tree Physiol. 27, 1261–1271. 10.1093/treephys/27.9.1261 17545126

[B33] Dardick C. (2007). Comparative expression profiling of Nicotiana benthamiana leaves systemically infected with three fruit tree viruses. Mol. Plant Microbe Interact. 20, 1004–1017. 10.1094/MPMI-20-8-1004 17722703

[B34] De Block M. Verduyn C. De Brouwer D. Cornelissen M. (2005). Poly(ADP-ribose) polymerase in plants affects energy homeostasis, cell death and stress tolerance. Plant J. 41, 95–106. 10.1111/j.1365-313X.2004.02277.x 15610352

[B35] de Carcer G. Medina F. J. (1999). Simultaneous localization of transcription and early processing markers allows dissection of functional domains in the plant cell nucleolus. J. Struct. Biol. 128, 139–151. 10.1006/jsbi.1999.4187 10600568

[B36] Degenhardt R. F. Bonham-Smith P. C. (2008). Arabidopsis ribosomal proteins RPL23aA and RPL23aB are differentially targeted to the nucleolus and are disparately required for normal development. Plant Physiol. 147, 128–142. 10.1104/pp.107.111799 18322146 PMC2330296

[B37] Donà M. Confalonieri M. Minio A. Biggiogera M. Buttafava A. Raimondi E.. (2013). RNA-Seq analysis discloses early senescence and nucleolar dysfunction triggered by Tdp1α depletion in *Medicago truncatula*. J. Exp. Bot. 64, 1941–1951. 10.1093/jxb/ert063 23467834

[B38] Donà M. Mittelsten Scheid O. (2015). DNA damage repair in the context of plant chromatin. Plant Physiol. 168, 1206–1218. 10.1104/pp.15.00538 26089404 PMC4528755

[B39] Dreyfuss G. Kim V. N. Kataoka N. (2002). Messenger-RNA-binding proteins and the messages they carry. Nat. Rev. Mol. Cell Biol. 3, 195–205. 10.1038/nrm760 11994740

[B40] Du Z. Chen A. Chen W. Liao Q. Zhang H. Bao Y.. (2014). Nuclear-cytoplasmic partitioning of cucumber mosaic virus protein 2b determines the balance between its roles as a virulence determinant and an RNA-silencing suppressor. J. Virol. 88, 5228–5541. 10.1128/JVI.00284-14 24599997 PMC4019134

[B41] Dundr M. Olson M. O. (1998). Partially processed pre-rRNA is preserved in association with processing components in nucleolus-derived foci during mitosis. Mol. Biol. Cell. 9, 2407–2422. 10.1091/mbc.9.9.2407 9725903 PMC25507

[B42] Dvoráčková M. Fojtová M. Fajkus J. (2015). Chromatin dynamics of plant telomeres and ribosomal genes. Plant J. 83, 18–37. 10.1111/tpj.12822 25752316

[B43] Dvorácková M. Rossignol P. Shaw P. J. Koroleva O. A. Doonan J. H. Fajkus J. (2010). AtTRB1, a telomeric DNA-binding protein from Arabidopsis, is concentrated in the nucleolus and shows highly dynamic association with chromatin. Plant J. 61, 637–649. 10.1111/j.1365-313X.2009.04094.x 19947985

[B44] Ender C. Krek A. Friedländer M. R. Beitzinger M. Weinmann L. Chen W.. (2008). A human snoRNA with microRNA-like functions. Mol. Cell. 32, 519–528. 10.1016/j.molcel.2008.10.017 19026782

[B45] Fromont-Racine M. Senger B. Saveanu C. Fasiolo F. (2003). Ribosome assembly in eukaryotes. Gene. 313, 17–42. 10.1016/S0378-1119(03)00629-2 12957375

[B46] Gerbi S. A. Borovjagin A. (1997). U3 snoRNA may recycle through different compartments of the nucleolus. Chromosoma 105, 401–406. 10.1007/BF02510476 9211967

[B47] Gilroy E. M. Breen S. Whisson S. C. Squires J. Hein I. Kaczmarek M.. (2011). Presence/absence, differential expression and sequence polymorphisms between PiAVR2 and PiAVR2-like in Phytophthora infestans determine virulence on R2 plants. New Phytol. 191, 763–776. 10.1111/j.1469-8137.2011.03736.x 21539575

[B48] Ginisty H. Amalric F. Bouvet P. (1998). Nucleolin functions in the first step of ribosomal RNA processing. EMBO J. 17, 1476–1486. 10.1093/emboj/17.5.1476 9482744 PMC1170495

[B49] Gong P. He C. (2014). Uncovering divergence of rice exon junction complex core heterodimer gene duplication reveals their essential role in growth, development, and reproduction. Plant Physiol. 165, 1047–1061. 10.1104/pp.114.237958 24820023 PMC4081321

[B50] González I. Martínez L. Rakitina D. V. Lewsey M. G. Atencio F. A. Llave C.. (2010). Cucumber mosaic virus 2b protein subcellular targets and interactions: their significance to RNA silencing suppressor activity. Mol. Plant Microbe Interact. 23, 294–303. 10.1094/MPMI-23-3-0294 20121451

[B51] González I. Rakitina D. Semashko M. Taliansky M. Praveen S. Palukaitis P.. (2012). RNA binding is more critical to the suppression of silencing function of Cucumber mosaic virus 2b protein than nuclear localization. RNA 18, 771–782. 10.1261/rna.031260.111 22357910 PMC3312564

[B52] González-Camacho F. Medina F. J. (2006). The nucleolar structure and the activity of NopA100, a nucleolin-like protein, during the cell cycle in proliferating plant cells. Histochem. Cell Biol. 125, 139–153. 10.1007/s00418-005-0081-1 16217651

[B53] Greco A. (2009). Involvement of the nucleolus in replication of human viruses. Rev. Med. Virol. 19, 201–214. 10.1002/rmv.614 19399920 PMC7169183

[B54] Grummt I. (2003). Life on a planet of its own: regulation of RNA polymerase I transcription in the nucleolus. Genes Dev. 17, 1691–1702. 10.1101/gad.1098503R 12865296

[B55] Hartung F. Suer S. Puchta H. (2007). Two closely related RecQ helicases have antagonistic roles in homologous recombination and DNA repair in *Arabidopsis thaliana*. Proc. Natl. Acad. Sci. U.S.A. 104, 18836–18841. 10.1073/pnas.0705998104 18000056 PMC2141863

[B56] Haupt S. Stroganova T. Ryabov E. Kim S. H. Fraser G. Duncan G.. (2005). Nucleolar localization of potato leafroll virus capsid proteins. J. Gen. Virol. 86, 2891–2896. 10.1099/vir.0.81101-0 16186245

[B57] He C. Sommer H. Grosardt B. Huijser P. Saedler H. (2007). PFMAGO, a MAGO NASHI-like factor, interacts with the MADS-domain protein MPF2 from Physalis floridana. Mol. Biol. Evol. 24, 1229–1241. 10.1093/molbev/msm041 17339635

[B58] Heliot L. Kaplan H. Lucas L. Klein C. Beorchia A. Doco-Fenzy M.. (1997). Electron tomography of metaphase nucleolar organizer regions: evidence for a twisted-loop organization. Mol. Biol. Cell. 8, 199–216. 10.1091/mbc.8.11.2199 9362063 PMC25702

[B59] Hernandez-Verdun D. (2011). Assembly and disassembly of the nucleolus during the cell cycle. Nucleus 2, 189–194. 10.4161/nucl.2.3.16246 21818412 PMC3149879

[B60] Herranz M. C. Pallas V. Aparicio F. (2012). Multifunctional roles for the N-terminal basic motif of Alfalfa mosaic virus coat protein: nucleolar/cytoplasmic shuttling, modulation of RNA-binding activity, and virion formation. Mol. Plant Microbe Interact. 25, 1093–1103. 10.1094/MPMI-04-12-0079-R 22746826

[B61] Hiscox J. A. (2007). RNA viruses: hijacking the dynamic nucleolus. Nat. Rev. Microbiol. 5, 119–127. 10.1038/nrmicro1597 17224921 PMC7097444

[B62] Ito T. Kim G. T. Shinozaki K. (2000). Disruption of an Arabidopsis cytoplasmic ribosomal protein S13-homologous gene by transposon mutagenesis causes aberrant growth and development. Plant J. 22, 257–264. 10.1046/j.1365-313x.2000.00728.x 10849343

[B63] Ji Y. Tulin A. V. (2013). Post-transcriptional regulation by poly(ADP-ribosyl)ation of the RNA-binding proteins. Int. J. Mol. Sci. 14, 16168–16183. 10.3390/ijms140816168 23921685 PMC3759905

[B64] Jones J. T. Kumar A. Pylypenko L. A. Thirugnanasambandam A. Castelli L. Chapman S.. (2009). Identification and functional characterization of effectors in expressed sequence tags from various life cycle stages of the potato cyst nematode *Globodera pallida*. Mol. Plant Pathol. 10, 815–828. 10.1111/j.1364-3703.2009.00585.x 19849787 PMC6640342

[B65] Khan A. Garbelli A. Grossi S. Florentin A. Batelli G. Acuna T.. (2014). The Arabidopsis STRESS RESPONSE SUPPRESSOR DEAD-box RNA helicases are nucleolar- and chromocenter-localized proteins that undergo stress-mediated relocalization and are involved in epigenetic gene silencing. Plant J. 79, 28–43. 10.1111/tpj.12533 24724701

[B66] Kim S. H. Koroleva O. A. Lewandowska D. Pendle A. F. Clark G. P. Simpson C. G.. (2009). Aberrant mRNA transcripts and the nonsense-mediated decay proteins UPF2 and UPF3 are enriched in the Arabidopsis nucleolus. Plant Cell. 21, 2045–2057. 10.1105/tpc.109.067736 19602621 PMC2729600

[B67] Kim S. H. Macfarlane S. Kalinina N. O. Rakitina D. V. Ryabov E. V. Gillespie T.. (2007a). Interaction of a plant virus-encoded protein with the major nucleolar protein fibrillarin is required for systemic virus infection. Proc. Natl. Acad. Sci. U.S.A. 104, 11115–11120. 10.1073/pnas.0704632104 17576925 PMC1904140

[B68] Kim S. H. Ryabov E. V. Kalinina N. O. Rakitina D. V. Gillespie T. MacFarlane S.. (2007b). Cajal bodies and the nucleolus are required for a plant virus systemic infection. EMBO J. 26, 2169–2179. 10.1038/sj.emboj.7601674 17410203 PMC1852794

[B69] Kim S. H. Spensley M. Choi S. K. Calixto C. P. Pendle A. F. Koroleva O.. (2010). Plant U13 orthologues and orphan snoRNAs identified by RNomics of RNA from Arabidopsis nucleoli. Nucleic Acids Res. 38, 3054–3067. 10.1093/nar/gkp1241 20081206 PMC2875012

[B70] Kim Y. K. Kim S. Shin Y. J. Hur Y. S. Kim W. Y. Lee M. S.. (2014). Ribosomal protein S6, a target of rapamycin, is involved in the regulation of rRNA genes by possible epigenetic changes in Arabidopsis. J. Biol. Chem. 289, 3901–3912. 10.1074/jbc.M113.515015 24302738 PMC3924259

[B71] Kojima H. Suzuki T. Kato T. Enomoto K. Sato S. Kato T.. (2007). Sugar-inducible expression of the nucleolin-1 gene of *Arabidopsis thaliana* and its role in ribosome synthesis, growth and development. Plant J. 49, 1053–1063. 10.1111/j.1365-313X.2006.03016.x 17286797

[B72] Koroleva O. A. Calder G. Pendle A. F. Kim S. H. Lewandowska D. Simpson C. G.. (2009). Dynamic behavior of Arabidopsis eIF4A-III, putative core protein of exon junction complex: fast relocation to nucleolus and splicing speckles under hypoxia. Plant Cell. 21, 1592–1606. 10.1105/tpc.108.060434 19435936 PMC2700535

[B73] Kotova E. Jarnik M. Tulin A. V. (2009). Poly (ADP-ribose) polymerase 1 is required for protein localization to Cajal body. PLoS Genet. 5:1000387. 10.1371/journal.pgen.1000387 19229318 PMC2637609

[B74] Lafontaine D. L. J. (2015). Noncoding RNAs in eukaryotic ribosome biogenesis and function. Nat. Struct. Mol. Biol. 22, 11–19. 10.1038/nsmb.2939 25565028

[B75] Lee S. Y. Kim H. Hwang H. J. Jeong Y. M. Na S. H. Woo J. C.. (2010). Identification of Tyrosyl-DNA phosphodiesterase as a novel DNA damage repair enzyme in Arabidopsis. Plant Physiol. 154, 1460–1469. 10.1104/pp.110.165068 20876339 PMC2971620

[B76] Leonelli L. Pelton J. Schoeffler A. Dahlbeck D. Berger J. Wemmer D. E.. (2011). Structural elucidation and functional characterization of the Hyaloperonospora arabidopsidis effector protein ATR13. PLoS Pathog. 7:1002428. 10.1371/journal.ppat.1002428 22194684 PMC3240608

[B77] Lewandowska D. ten Have S. Hodge K. Tillemans V. Lamond A. I. Brown J. W. (2013). Plant SILAC: stable-isotope labelling with amino acids of arabidopsis seedlings for quantitative proteomics. PLoS ONE 8:72207. 10.1371/journal.pone.0072207 23977254 PMC3748079

[B78] Li C. Zhang B. (2016). MicroRNAs in control of plant development. J. Cell. Physiol. 231, 303–313. 10.1002/jcp.25125 26248304

[B79] Li W. Zhao Y. Liu C. Yao G. Wu S. Hou C.. (2012). Callose deposition at plasmodesmata is a critical factor in restricting the cell-to-cell movement of Soybean mosaic virus. Plant Cell Rep. 31, 905–916. 10.1007/s00299-011-1211-y 22200865

[B80] Li Z. Zhang Y. Jiang Z. Jin X. Zhang K. Wang X.. (2017). Hijacking of the nucleolar protein fibrillarin by TGB1 is required for cell-to-cell movement of *Barley stripe mosaic* virus. Mol. Plant Pathol. 10.1111/mpp.12612 28872759 PMC6638131

[B81] Lindström M. S. (2009). Emerging functions of ribosomal proteins in gene-specific transcription and translation. Biochem. Biophys. Res. Commun. 379, 167–170. 10.1016/j.bbrc.2008.12.083 19114035

[B82] Lorković Z. J. Barta A. (2008). Role of Cajal bodies and nucleolus in the maturation of the U1 snRNP in Arabidopsis. PLoS ONE 3:3989. 10.1371/journal.pone.0003989 19098980 PMC2600615

[B83] Lough T. J. Lucas W. J. (2006). Integrative plant biology: role of phloem long-distance macromolecular trafficking. Annu. Rev. Plant Biol. 57, 203–232. 10.1146/annurev.arplant.56.032604.144145 16669761

[B84] Love A. J. Yu C. Petukhova N. V. Kalinina N. O. Chen J. Taliansky M. E. (2017). Cajal bodies and their role in plant stress and disease responses. RNA Biol. 14, 779–790. 10.1080/15476286.2016.1243650 27726481 PMC5519230

[B85] Luo X. Kraus W. L. (2012). On PAR with PARP: cellular stress signaling through poly(ADP-ribose) and PARP-1. Genes Dev. 26, 417–432. 10.1101/gad.183509.111 22391446 PMC3305980

[B86] Ma H. Song T. Wang T. Wang S. (2016). Influence of human p53 on plant development. PLoS ONE 11:e0162840. 10.1371/journal.pone.0162840 27648563 PMC5029891

[B87] Mais C. Wright J. E. Prieto J. L. Raggett S. L. McStay B. (2005). UBF-binding site arrays form pseudo-NORs and sequester the RNA polymerase I transcription machinery. Genes Dev. 19, 50–64. 10.1101/gad.310705 15598984 PMC540225

[B88] Mallory A. Vaucheret H. (2010). Form, function, and regulation of ARGONAUTE proteins. Plant Cell. 22, 3879–3889. 10.1105/tpc.110.080671 21183704 PMC3027166

[B89] Manova V. Gruszka D. (2015). DNA damage and repair in plants–from models to crops. Front. Plant Sci. 6:885. 10.3389/fpls.2015.00885 26557130 PMC4617055

[B90] Maquat L. E. (2004). Nonsense-mediated mRNA decay: splicing, translation and mRNP dynamics. Nat. Rev. Mol. Cell Biol. 5, 89–99. 10.1038/nrm1310 15040442

[B91] Matera A. G. Terns R. M. Terns M. P. (2007). Non-coding RNAs: lessons from the small nuclear and small nucleolar RNAs. Nat. Rev. Mol. Cell Biol. 8, 209–220. 10.1038/nrm2124 17318225

[B92] McKeown P. C. Shaw P. J. (2009). Chromatin: linking structure and function in the nucleolus. Chromosoma 118, 11–23. 10.1007/s00412-008-0184-2 18925405

[B93] Mineur P. Jennane A. Thiry M. Deltour R. Goessens G. (1998). Ultrastructural distribution of DNA within plant meristematic cell nucleoli during activation and the subsequent inactivation by a cold stress. J. Struct. Biol. 123, 199–210. 10.1006/jsbi.1998.4038 9878575

[B94] Murano K. Okuwaki M. Hisaoka M. Nagata K. (2008). Transcription regulation of the rRNA gene by a multifunctional nucleolar protein, B23/nucleophosmin, through its histone chaperone activity. Mol. Cell. Biol. 28, 3114–3126. 10.1128/MCB.02078-07 18332108 PMC2423177

[B95] Nazar R. N. (2004). Ribosomal RNA processing and ribosome biogenesis in eukaryotes. IUBMB Life 56, 457–465. 10.1080/15216540400010867 15545225

[B96] Nechushtai R. Conlan A. R. Harir Y. Song L. Yogev O. Eisenberg-Domovich Y.. (2012). Characterization of Arabidopsis NEET reveals an ancient role for NEET proteins in iron metabolism. Plant Cell. 24, 2139–2154. 10.1105/tpc.112.097634 22562611 PMC3442592

[B97] Nishimura T. Wada T. Yamamoto K. T. Okada K. (2005). TheArabidopsis STV1 protein, responsible for translation reinitiation, is required for auxin-mediated gynoecium patterning. Plant Cell. 17, 2940–2953. 10.1105/tpc.105.036533 16227452 PMC1276021

[B98] Ohtani M. Demura T. Sugiyama M. (2013). Arabidopsis root initiation defective1, a DEAH-box RNA helicase involved in pre-mRNA splicing, is essential for plant development. Plant Cell. 25, 2056–2069. 10.1105/tpc.113.111922 23771891 PMC3723612

[B99] Olson M. O. Dundr M. (2005). The moving parts of the nucleolus. Histochem. Cell Biol. 123, 203–216. 10.1007/s00418-005-0754-9 15742198

[B100] Ono M. Scott M. S. Yamada K. Avolio F. Barton G. J. Lamond A. I. (2011). Identification of human miRNA precursors that resemble box C/D snoRNAs. Nucleic Acids Res. 39, 3879–3891. 10.1093/nar/gkq1355 21247878 PMC3089480

[B101] Park N. I. Yeung E. C. Muench D. G. (2009). Mago Nashi is involved in meristem organization, pollen formation, and seed development in Arabidopsis. Plant Sci. 176, 461–469. 10.1016/j.plantsci.2008.12.016 26493135

[B102] Pederson T. (1998). The plurifunctional nucleolus. Nucleic Acids Res. 26, 3871–3876. 10.1093/nar/26.17.3871 9705492 PMC147800

[B103] Pendle A. F. Clark G. P. Boon R. Lewandowska D. Lam Y. W. Andersen J.. (2005). Proteomic analysis of the Arabidopsis nucleolus suggests novel nucleolar functions. Mol. Biol. Cell. 16, 260–269. 10.1091/mbc.E04-09-0791 15496452 PMC539170

[B104] Petre B. Saunders D. G. Sklenar J. Lorrain C. Win J. Duplessis S.. (2015). Candidate effector proteins of the rust pathogen melampsora larici-populina target diverse plant cell compartments. Mol. Plant Microbe Interact. 28, 689–700. 10.1094/MPMI-01-15-0003-R 25650830

[B105] Politz J. C. Hogan E. M. Pederson T. (2009). MicroRNAs with a nucleolar location. RNA 15, 1705–1715. 10.1261/rna.1470409 19628621 PMC2743059

[B106] Pontes O. Li C. F. Costa Nunes P. Haag J. Ream T. Vitins A.. (2006). The Arabidopsis chromatin-modifying nuclear siRNA pathway involves a nucleolar RNA processing center. Cell 126, 79–92. 10.1016/j.cell.2006.05.031 16839878

[B107] Pontes O. Pikaard C. S. (2008). siRNA and miRNA processing: new functions for Cajal bodies. Curr. Opin. Genet. Dev. 18, 197–203. 10.1016/j.gde.2008.01.008 18337083 PMC2483300

[B108] Pontes O. Vitins A. Ream T. S. Hong E. Pikaard C. S. Costa-Nunes P. (2013). Intersection of small RNA pathways in *Arabidopsis thaliana* sub-nuclear domains. PLoS ONE 8:65652. 10.1371/journal.pone.0065652 23776518 PMC3680462

[B109] Pontvianne F. Abou-Ellail M. Douet J. Comella P. Matia I. Chandrasekhara C.. (2010). Nucleolin is required for DNA methylation state and the expression of rRNA gene variants in *Arabidopsis thaliana*. PLoS Genet. 6:1001225. 10.1371/journal.pgen.1001225 21124873 PMC2991258

[B110] Pontvianne F. Carpentier M. C. Durut N. Pavlištová V. Jaške K. Schorová Š.. (2016). Identification of nucleolus-associated chromatin domains reveals a role for the nucleolus in 3D organization of the *A. thaliana* genome. Cell Rep. 16, 1574–1587. 10.1016/j.celrep.2016.07.016 27477271 PMC5279810

[B111] Pontvianne F. Matía I. Douet J. Tourmente S. Medina F. J. Echeverria M.. (2007). Characterization of AtNUC-L1 reveals a central role of nucleolin in nucleolus organization and silencing of AtNUC-L2 gene in Arabidopsis. Mol. Biol. Cell. 18, 369–379. 10.1091/mbc.E06-08-0751 17108323 PMC1783796

[B112] Prieto J. L. McStay B. (2008). Pseudo-NORs: a novel model for studying nucleoli. Biochim. Biophys. Acta. 1783, 2116–2123. 10.1016/j.bbamcr.2008.07.004 18687368

[B113] Procházková Schrumpfová P. Schorová Š. Fajkus J. (2016). Telomere- and telomerase-associated proteins and their functions in the plant cell. Front. Plant Sci. 7:851. 10.3389/fpls.2016.00851 27446102 PMC4924339

[B114] Rajamäki M. L. Valkonen J. P. (2009). Control of nuclear and nucleolar localization of nuclear inclusion protein a of picorna-like Potato virus A in Nicotiana species. Plant Cell. 21, 2485–2502. 10.1105/tpc.108.064147 19700632 PMC2751958

[B115] Rajamäki M. L. Xi D. Sikorskaite-Gudziuniene S. Valkonen J. P. T. Whitham S. A. (2017). Differential requirement of the Ribosomal Protein S6 and Ribosomal Protein S6 kinase for Plant-Virus accumulation and interaction of S6 kinase with Potyviral VPg. Mol. Plant Microbe Interact. 30, 374–384. 10.1094/MPMI-06-16-0122-R 28437137

[B116] Renák D. Gibalová A. Solcová K. Honys D. (2014). A new link between stress response and nucleolar function during pollen development in Arabidopsis mediated by AtREN1 protein. Plant Cell Environ. 37, 670–683. 10.1111/pce.12186 23961845

[B117] Ren S. Mandadi K. K. Boedeker A. L. Rathore K. S. McKnight T. D. (2007). Regulation of telomerase in Arabidopsis by BT2, an apparent target of TELOMERASE ACTIVATOR1. Plant Cell. 19, 23–31. 10.1105/tpc.106.044321 17220202 PMC1820974

[B118] Roger B. Moisand A. Amalric F. Bouvet P. (2003). Nucleolin provides a link between RNA polymerase I transcription and pre-ribosome assembly. Chromosoma 111, 399–407. 10.1007/s00412-002-0221-5 12644954

[B119] Röhrig S. Schröpfer S. Knoll A. Puchta H. (2016). The RTR complex partner RMI2 and the DNA Helicase RTEL1 are both independently involved in preserving the stability of 45S rDNA repeats in *Arabidopsis thaliana*. PLoS Genet. 12:e1006394. 10.1371/journal.pgen.1006394 27760121 PMC5070779

[B120] Russell J. Zomerdijk J. C. (2005). RNA-polymerase-I-directed rDNA transcription, life and works. Trends Biochem. Sci. 30, 87–96. 10.1016/j.tibs.2004.12.008 15691654 PMC3858833

[B121] Saraiya A. A. Wang C. C. (2008). snoRNA, a novel precursor of microRNA in *Giardia lamblia*. PLoS Pathog. 4:1000224. 10.1371/journal.ppat.1000224 19043559 PMC2583053

[B122] Sato S. Yano H. Makimoto Y. Kaneta T. Sato Y. (2005). Nucleolonema as a fundamental substructure of the nucleolus. J. Plant Res. 118, 71–81. 10.1007/s10265-005-0204-8 15843864

[B123] Schulz P. Jansseune K. Degenkolbe T. Méret M. Claeys H. Skirycz A.. (2014). Poly(ADP-ribose)polymerase activity controls plant growth by promoting leaf cell number. PLoS ONE 9:90322. 10.1371/journal.pone.0090322 24587323 PMC3938684

[B124] Scott M. S. Avolio F. Ono M. Lamond A. I. Barton G. J. (2009). Human miRNA precursors with box H/ACA snoRNA features. PLoS Comput. Biol. 5:1000507. 10.1371/journal.pcbi.1000507 19763159 PMC2730528

[B125] Semashko M. A. González I. Shaw J. Leonova O. G. Popenko V. I. Taliansky M. E.. (2012). The extreme N-terminal domain of a hordeivirus TGB1 movement protein mediates its localization to the nucleolus and interaction with fibrillarin. Biochimie 94, 1180–1188. 10.1016/j.biochi.2012.02.005 22349738

[B126] Shaw J. Love A. J. Makarova S. S. Kalinina N. O. Harrison B. D. Taliansky M. E. (2014). Coilin, the signature protein of Cajal bodies, differentially modulates the interactions of plants with viruses in widely different taxa. Nucleus 5, 85–94. 10.4161/nucl.28315 24637832 PMC4028359

[B127] Shaw P. Brown J. (2012). Nucleoli: composition, function, and dynamics. Plant Physiol. 158, 44–51. 10.1104/pp.111.188052 22082506 PMC3252080

[B128] Shaw P. J. (1996). Nuclear organization in plants. Essays Biochem. 31, 77–89. 9078459

[B129] Shaw P. J. (2015). ≪Nucleolus≫, in Encyclopedia of Life Sciences. Chichester: John Wiley and Sons, Ltd, 17080–17088.

[B130] Shaw P. J. Brown J. W. (2004). Plant nuclear bodies. Curr. Opin. Plant Biol. 7, 614–620. 10.1016/j.pbi.2004.09.011 15491908

[B131] Shaw P. J. Highett M. I. Beven A. F. Jordan E. G. (1995). The nucleolar architecture of polymerase I transcription and processing. EMBO J. 14, 2896–2906. 7796815 10.1002/j.1460-2075.1995.tb07289.x PMC398408

[B132] Shaw P. J. Jordan E. G. (1995). The nucleolus. Annu. Rev. Cell Dev. Biol. 11, 93–121. 10.1146/annurev.cb.11.110195.000521 8689574

[B133] Shi Y. Liu X. Li R. Gao Y. Xu Z. Zhang B.. (2014). Retention of OsNMD3 in the cytoplasm disturbs protein synthesis efficiency and affects plant development in rice. J. Exp. Bot. 65, 3055–3069. 10.1093/jxb/eru150 24723395 PMC4071826

[B134] Sirri V. Urcuqui-Inchima S. Roussel P. Hernandez-Verdun D. (2008). Nucleolus: the fascinating nuclear body. Histochem. Cell Biol. 129, 13–31. 10.1007/s00418-007-0359-6 18046571 PMC2137947

[B135] Son O. Kim S. Hur Y. S. Cheon C. I. (2016). Identification of the Raptor-binding motif on Arabidopsis S6 kinase and its use as a TOR signaling suppressor. Biochem. Biophys. Res. Commun. 472, 83–87. 10.1016/j.bbrc.2016.02.068 26920057

[B136] Son O. Kim S. Shin Y. J. Kim W. Y. Koh H. J. Cheon C. I. (2015). Identification of nucleosome assembly protein 1 (NAP1) as an interacting partner of plant ribosomal protein S6 (RPS6) and a positive regulator of rDNA transcription. Biochem. Biophys. Res. Commun. 465, 200–205. 10.1016/j.bbrc.2015.07.150 26241676

[B137] Stam R. Howden A. J. Delgado-Cerezo M. M. M. Amaro T. M. Motion G. B. Pham J.. (2013). Characterization of cell death inducing Phytophthora capsici CRN effectors suggests diverse activities in the host nucleus. Front. Plant Sci. 4:387. 10.3389/fpls.2013.00387 24155749 PMC3803116

[B138] Stepinski D. (2009). Immunodetection of nucleolar proteins and ultrastructure of nucleoli of soybean root meristematic cells treated with chilling stress and after recovery. Protoplasma 235, 77–89. 10.1007/s00709-009-0033-z 19241118

[B139] Stepinski D. (2012). Immunofluorescent localization of ubiquitin and proteasomes in nucleolar vacuoles of soybean root meristematic cells. Eur. J. Histochem. 56:13. 10.4081/ejh.2012.e13 22688294 PMC3428962

[B140] Stepinski D. (2014). Functional ultrastructure of the plant nucleolus. Protoplasma 251, 1285–1306. 10.1007/s00709-014-0648-6 24756369 PMC4209244

[B141] Taft R. J. Glazov E. A. Lassmann T. Hayashizaki Y. Carninci P. Mattick J. S. (2009). Small RNAs derived from snoRNAs. RNA 15, 1233–1240. 10.1261/rna.1528909 19474147 PMC2704076

[B142] Taliansky M. E. Brown J. W. Rajamäki M. L. Valkonen J. P. Kalinina N. O. (2010). Involvement of the plant nucleolus in virus and viroid infections: parallels with animal pathosystems. Adv. Virus Res. 77, 119–158. 10.1016/B978-0-12-385034-8.00005-3 20951872 PMC7149663

[B143] Tian R. H. Zhang G. Y. Yan C. H. Dai Y. R. (2000). Involvement of poly(ADP-ribose) polymerase and activation of caspase-3-like protease in heat shock-induced apoptosis in tobacco suspension cells. FEBS Lett. 474, 11–15. 10.1016/S0014-5793(00)01561-1 10828442

[B144] Toledo-Ortiz G. Huq E. Quail P. H. (2003). The Arabidopsis basic/helix-loop-helix transcription factor family. Plant Cell. 15, 1749–1770. 10.1105/tpc.013839 12897250 PMC167167

[B145] Tollervey D. Lehtonen H. Jansen R. Kern H. Hurt E. C. (1993). Temperature-sensitive mutations demonstrate roles for yeast fibrillarin in pre-rRNA processing, pre-rRNA methylation, and ribosome assembly. Cell 72, 443–457. 10.1016/0092-8674(93)90120-F 8431947

[B146] Van Lijsebettens M. Vanderhaeghen R. De Block M. Bauw G. Villarroel R. Van Montagu M. (1994). An S18 ribosomal protein gene copy at the Arabidopsis PFL locus affects plant development by its specific expression in meristems. EMBO J. 13, 3378–3388. 7913892 10.1002/j.1460-2075.1994.tb06640.x PMC395235

[B147] Vaucheret H. (2008). Plant ARGONAUTES. Trends Plant Sci. 13, 350–358. 10.1016/j.tplants.2008.04.007 18508405

[B148] Warner J. R. (1990). The nucleolus and ribosome formation. Curr. Opin. Cell Biol. 2, 521–527. 10.1016/0955-0674(90)90137-4 2198902

[B149] Weijers D. Franke-van Dijk M. Vencken R. J. Quint A. Hooykaas P. Offringa R. (2001). An Arabidopsis Minute-like phenotype caused by a semi-dominant mutation in a RIBOSOMAL PROTEIN S5 gene. Development 128, 4289–4299. 11684664 10.1242/dev.128.21.4289

[B150] Weis B. L. Palm D. Missbach S. Bohnsack M. T. Schleiff E. (2015a). atBRX1-1 and atBRX1-2 are involved in an alternative rRNA processing pathway in *Arabidopsis thaliana*. RNA 21, 415–425. 10.1261/rna.047563.114 25605960 PMC4338337

[B151] Weis B. L. Kovacevic J. Missbach S. Schleiff E. (2015b). Plant-specific features of ribosome biogenesis. Trends Plant Sci. 20, 729–740. 10.1016/j.tplants.2015.07.003 26459664

[B152] Xiong Y. Sheen J. (2014). The role of target of rapamycin signaling networks in plant growth and metabolism. Plant Physiol. 164, 499–512. 10.1104/pp.113.229948 24385567 PMC3912084

[B153] Yang C. Guo R. Jie F. Nettleton D. Peng J. Carr T.. (2007). Spatial analysis of *Arabidopsis thaliana* gene expression in response to *Turnip mosaic* virus infection. Mol. Plant Microbe Interact. 20, 358–370. 10.1094/MPMI-20-4-0358 17427806

[B154] Yang C. Zhang C. Dittman J. D. Whitham S. A. (2009). Differential requirement of ribosomal protein S6 by plant RNA viruses with different translation initiation strategies. Virology 390, 163–173. 10.1016/j.virol.2009.05.018 19524993

[B155] Yang S. W. Jin E. Chung I. K. Kim W. T. (2002). Cell cycle-dependent regulation of telomerase activity by auxin, abscisic acid and protein phosphorylation in tobacco BY-2 suspension culture cells. Plant J. 29, 617–626. 10.1046/j.0960-7412.2001.01244.x 11874574

[B156] Yang Z. P. Li H. L. Guo D. Peng S. Q. (2016). Identification and characterization of MAGO and Y14 genes in Hevea brasiliensis. Genet. Mol. Biol. 39, 73–85. 10.1590/1678-4685-GMB-2014-0387 27007901 PMC4807384

[B157] Yano H. Sato S. (2002). Combination of electron microscopic in situ hybridization and anti-DNA antibody labelling reveals a peculiar arrangement of ribosomal DNA in the fibrillar centres of the plant cell nucleolus. J. Electron Microsc. (Tokyo). 51, 231–239. 10.1093/jmicro/51.4.231 12227553

[B158] Yoshiyama K. O. (2016). SOG1: a master regulator of the DNA damage response in plants. Genes Genet. Syst. 90, 209–216. 10.1266/ggs.15-00011 26617076

[B159] Yoshiyama K. O. Kimura S. Maki H. Britt A. B. Umeda M. (2014). The role of SOG1, a plant-specific transcriptional regulator, in the DNA damage response. Plant Signal. Behav. 9:e28889. 10.4161/psb.28889 24736489 PMC4091597

[B160] Zhang S. Sun L. Kragler F. (2009). The phloem-delivered RNA pool contains small non-coding RNAs and interferes with translation. Plant Physiol. 150, 378–387. 10.1104/pp.108.134767 19261735 PMC2675743

[B161] Zheng L. Du Z. Lin C. Mao Q. Wu K. Wu J.. (2015). Rice stripe tenuivirus p2 may recruit or manipulate nucleolar functions through an interaction with fibrillarin to promote virus systemic movement. Mol. Plant Pathol. 16, 921–930. 10.1111/mpp.12220 25431002 PMC6638460

